# Genetic Diversity of Nitrogen-Fixing and Plant Growth Promoting *Pseudomonas* Species Isolated from Sugarcane Rhizosphere

**DOI:** 10.3389/fmicb.2017.01268

**Published:** 2017-07-14

**Authors:** Hai-Bi Li, Rajesh K. Singh, Pratiksha Singh, Qi-Qi Song, Yong-Xiu Xing, Li-Tao Yang, Yang-Rui Li

**Affiliations:** ^1^Agricultural College, State Key Laboratory of Subtropical Bioresources Conservation and Utilization, Guangxi University Nanning, China; ^2^Key Laboratory of Sugarcane Biotechnology and Genetic Improvement Guangxi, Ministry of Agriculture, Sugarcane Research Center, Chinese Academy of Agricultural Sciences, Sugarcane Research Institute, Guangxi Academy of Agricultural Sciences Nanning, China

**Keywords:** antibiotic gene, genetic diversity, GFP, *Pseudomonas*, *nifH*, sugarcane, Biolog

## Abstract

The study was designed to isolate and characterize *Pseudomonas* spp. from sugarcane rhizosphere, and to evaluate their plant- growth- promoting (PGP) traits and nitrogenase activity. A biological nitrogen-fixing microbe has great potential to replace chemical fertilizers and be used as a targeted biofertilizer in a plant. A total of 100 isolates from sugarcane rhizosphere, belonging to different species, were isolated; from these, 30 isolates were selected on the basis of preliminary screening, for *in vitro* antagonistic activities against sugarcane pathogens and for various PGP traits, as well as nitrogenase activity. The production of IAA varied from 312.07 to 13.12 μg mL^−1^ in tryptophan supplemented medium, with higher production in AN15 and lower in CN20 strain. The estimation of ACC deaminase activity, strains CY4 and BA2 produced maximum and minimum activity of 77.0 and 15.13 μmoL mg^−1^ h^−1^. For nitrogenase activity among the studied strains, CoA6 fixed higher and AY1 fixed lower in amounts (108.30 and 6.16 μmoL C_2_H_2_ h^−1^ mL^−1^). All the strains were identified on the basis of 16S rRNA gene sequencing, and the phylogenetic diversity of the strains was analyzed. The results identified all strains as being similar to *Pseudomonas* spp. Polymerase chain reaction (PCR) amplification of *nifH* and antibiotic genes was suggestive that the amplified strains had the capability to fix nitrogen and possessed biocontrol activities. Genotypic comparisons of the strains were determined by BOX, ERIC, and REP PCR profile analysis. Out of all the screened isolates, CY4 (*Pseudomonas koreensis*) and CN11 (*Pseudomonas entomophila*) showed the most prominent PGP traits, as well as nitrogenase activity. Therefore, only these two strains were selected for further studies; Biolog profiling; colonization through green fluorescent protein (GFP)-tagged bacteria; and *nifH* gene expression using quantitative real-time polymerase chain reaction (qRT-PCR) analysis. The Biolog phenotypic profiling, which comprised utilization of C and N sources, and tolerance to osmolytes and pH, revealed the metabolic versatility of the selected strains. The colonization ability of the selected strains was evaluated by genetically tagging them with a constitutively expressing GFP-pPROBE-pTet^r^-OT plasmid. qRT-PCR results showed that both strains had the ability to express the *nifH* gene at 90 and 120 days, as compared to a control, in both sugarcane varieties GT11 and GXB9. Therefore, our isolated strains, *P. koreensis* and *P. entomophila* may be used as inoculums or in biofertilizer production for enhancing growth and nutrients, as well as for improving nitrogen levels, in sugarcane and other crops. The present study, to the best of our knowledge, is the first report on the diversity of *Pseudomonas* spp. associated with sugarcane in Guangxi, China.

## Introduction

Sugarcane (*Saccharum officinarum* L.) is one of the most important industrial agricultural crops, being cultivated in over 110 tropical and subtropical countries and, providing a source of sugar, renewable energy, and biomaterials (Fischer et al., 2012). The main sugarcane producing country in the world market is Brazil, and the next major producers are India, China and Thailand (FAO, 2016). More than fifty diseases are caused by plant pathogens in sugarcane (Croft and Magarey, 2000; Rao et al., 2002) with 10–15% of sugar being lost due to such diseases. Among them, red rot, smut, wilt, and pineapple diseases caused by fungi, and ratoon stunting disease caused by bacteria, are found to cause considerable yield loss (Viswanathan and Rao, 2011). Sugarcane is a long duration economical crop, so it requires large amounts of plant nutrients i.e., N, P, K, as well as of other micro nutrients. An abundant supply of nitrogen is required for the early stages of plant growth. However, in many countries, farmers apply even higher doses of fertilizers, chemicals, and pesticides to sugarcane to promote early growth and development and to increase yields. Although, N fertilizer use is comparatively low in Brazil (~50 kg N ha^−1^), other countries average ~120–300 kg N ha^−1^ with extreme rates in excess of 700 kg N ha^−1^ (Robinson et al., 2011). However, a higher dose of fertilizer not only raises the production cost, but also causes serious environmental pollution (Herridge et al., 2008; Li and Yang, 2015). It may have negative and unpredictable effects on the environment, and contribute to the pollution of soil, water, and natural areas.

There is vast microbial flora available globally, and microbes are found in all types of soils, such as sands, deserts, and soils of volcanic origin, and in bogs and moors, snow covered soils, sediments, and semi-aquatic ecosystems, and on rocks (Manoharachary and Mukerji, 2006). There is a clear incentive to exploit this microbial diversity and to isolate and develop functional microbes that can be used, in effect, as targeted fertilizers as an alternative to traditional fertilizer applications. Here, we focused on nitrogen-fixing bacterial genera that are often found in large populations in rhizospheric soils and that exhibit general disease-suppression and PGP traits. In principle, biological nitrogen fixation (BNF) promises an alternative approach to plant N fertilizer requirements (Xing et al., 2006, 2015). Some Brazilian sugarcane varieties are capable of obtaining substantial nitrogen from the soil through BNF (Lima et al., 1987; Urquiaga et al., 1992, 2012). Studies using long-term N balances, ^15^N natural abundance, and ^15^N isotope dilution methods have shown that some sugarcane cultivars can obtain a significant amount of their nitrogen requirements in this way (Urquiaga et al., 1992, 2012), but the bacteria responsible remains unknown (Boddey et al., 1995; James and Olivares, 1998; James, 2000).

A diverse array of bacteria, including species of Azoarcus, Azospirillum, Arthrobacter, Azotobacter, Bacillus, Burkholderia, Erwinia, Enterobacter, Gluconacetobacter, Herbaspirillum seropedicae, Klebsiella, Kosakonia, Paenibacillus, Pantoea, Pseudomonas, Stenotrophomonas, Serratia, and Xanthomonas are among the main plant growth promoting rhizobacteria used to promote the growth of several crops, including sugarcane (Somers et al., 2004; Bhattacharyya and Jha, 2012; Carvalho et al., 2014; Rafikova et al., 2016; Xing et al., 2016; Solanki et al., 2017). Strains of some bacterial genera, e.g., Azotobacter, Bacillus, Enterobacter, Pseudomonas, Serratia, and Azospirillum are already being used as biofertilizers for enhancing the growth and yield of crops, as well as for maintaining soil fertility (De Souza et al., 2015). Nitrogen-fixing microorganisms play an important role both in the soil and in plants. Many plant-associated rhizobacteria are recognized for their PGP ability, for their capacity to increase disease resistance, and for their of phytohormones under various stress conditions. However, the use of inoculated microbial activity requires observation of the efficiency and colonization rate to track and identify the inoculated strain within the host plant. We used a popular marker gene that encodes green fluorescent protein (GFP), which was easily detected in cell samples by using confocal microscopy (Unge et al., 1999). Confocal laser scanning electron microscopy (CLSEM), in combination with GFP is a powerful tool for studying plant-microbe interactions (Chi et al., 2004; Liu et al., 2006). N_2_-fixing microorganisms contain dinitrogenase, one of the subunits of which is encoded by nifH, the detection of nifH mRNA indicates the presence of N_2_-fixing bacteria, as well as indicating N_2_-fixation in plants (Young, 1992). Quantitative real-time polymerase chain reaction (qRT-PCR) has been found to be a powerful approach for quantification of an active N_2_-fixing population within a multifarious community (Wallenstein, 2004). The undeviating contribution of plant-inhabiting diazotrophs (*Azospirillum* spp., *Rhizobium* spp., etc.) to nitrogen-fixation and nitrogen-uptake in cucumber has been estimated by quantifying the nifH gene copy number using qRT-PCR (Juraeva et al., 2006).

Researchers have explored and focused on identifying N_2_-fixing *Pseudomonas* associated with plants to increase crop production, reduce harmful chemicals and protect the soil and environment. *Pseudomonas* spp. belong to the family *Pseudomonadaceae*, which contains a large number of species and is divided into subdivisions (Mehnaz, 2011). *Pseudomonas fluorescens* and *Pseudomonas putida* are very well-known and well-studied species of this genus that have been used as inoculums to promote plant growth. Some earlier reports on the isolation of *Pseudomonas* from sugarcane are: *Pseudomonas* spp. (Li and Macrae, 1991; Antwerpen et al., 2002; Magnani et al., 2010), *P. aeruginosa* (Viswanathan et al., 2003), *P. aurantia*ca (Mehnaz et al., 2009b), *P. fluorescens* (Viswanathana and Samiyappan, 2002; Mendes et al., 2007; Mehnaz et al., 2009a), *P. putida* (Viswanathana and Samiyappan, 2002; Mehnaz et al., 2009a), and *P. reactans* (Mehnaz et al., 2010).

In this study, *Pseudomonas* strains were isolated from the rhizosphere of sugarcane plants grown in the field in Guangxi, China. We specifically focused on the diversity of *Pseudomonas* spp. and the major objectives were: (1) to investigate the antagonistic ability of *Pseudomonas* spp. isolated from sugarcane against pathogens; (2) to evaluate their PGP traits as well as nitrogenase activities in order to use them further as bio-fertilizers; (3) to use polymerase chain reaction (PCR) and qRT-PCR based techniques to detect the *nifH* gene, characterize antibiotic genes, and assess their genetic diversity through BOX, ERIC and REP-PCR; (4) to analyse 16S rRNA gene sequences for effective identification of *Pseudomonas* spp.; (5) to test the utilization of numerous sources of carbon, and nitrogen, as well as tolerance to osmolytes and different pH conditions and (6) to investigate the interaction mechanisms between the sugarcane plant and selected potential strains through a GFP technique. As for the available literature, to the best of our knowledge, this is the first report on nitrogen-fixing *Pseudomonas koreensis* and *Pseudomonas entomophila* isolated from sugarcane in China.

## Materials and methods

### Locations and collection of soil sampling site and properties

The study area is Nanning City, Guangxi Autonomous Region in South China. It has a warm with an average temperature of 21.7°C and humid subtropical climate. Summers are hot and the average temperature is 25 (lowest), 33°C (highest) in July, and winters are the coldest, 10°C in January. The average annual rainfall is between 1,000–2,800 mm and precipitation is 1,372 mm. It is situated between 22°49′1.21″ N latitude and 108°21′59.55″ E longitude, and elevation is 79.51 m.

Soil samples were randomly collected from sugarcane fields that have highly fertile soils. Five healthy plants were sampled using a sterile auger from different locations in a sterile specimen container and immediately transported to the laboratory. In all cases, soil samples were taken from 2 to 20 cm layers in April 2015. The soil particles attached to roots were carefully collected after uprooting plants and mixed well. Root debris was removed by sieving through 2 mm mesh. Samples were stored at 4°C for further studies and processed within 24 h of collection. The pulverized soil samples were used for analysis of physico-chemical properties.

### Media and growth conditions of bacterial strains

We selected four different enrichment media (Ashbey's medium, Yeast Mannitol Agar, LGI, and Dworkin and Foster salts minimal medium) for the isolation of bacteria from soil samples; all the media contained some component that permitted the growth of specific types of nitrogen-fixing bacteria. A universal nutrient agar (NA) medium was also used for the isolation of all types of bacterial strains (Table S1). Ten grams of soil from each sample were separately suspended in 90 mL of saline water (0.85% of NaCl) in a flask and placed on an orbital shaker (at 100 rpm) at 30 ± 2°C for 1 h.

### *In vitro* test of plant-growth-promoting attributes

The growth promotion traits of all bacterial isolates were evaluated by performing standard protocol for the estimation of indole acetic acid (IAA), P-solubilization, siderophore, hydrogen cyanide (HCN) and ammonia production according to Glickmann and Dessaux (1995), Brick et al. (1991), Schwyn and Neilands (1987), Lorck (1948) and Dey et al. (2004), respectively. For P-solubilization, plates containing Pikovskaya's media amended with tri-calcium phosphate were observed for clearing or solubilisation zones around the colonies. For siderophore production the Chrome Azurol S (CAS) medium was prepared by mixing 25 mL of solution A (composition in gL^−1^: 60.5 mg CAS was dissolved in 50 mL of distilled water and 10 mL iron (III) solution; 1 mM FeCl_3_.6H_2_O, 10 mm HCl). This solution was slowly added to 72.9 mg hexadecyl-trimethyl ammonium bromide (HDTMA) dissolved in 40 mL of water. The resultant dark medium was autoclaved and 75 mL of solution B (Nutrient agar) after autoclaving separately mixed at about 40–50°C and chromazurol sulphonate agar plate has been prepared. The bacterial strains were spot inoculated on chromazurol sulphonate agar plate medium and incubated at 28 ± 2°C for 2–6 days. After incubation of plates siderophore production was assayed by the change in the color of the medium from blue to orange haloes zone formation.

### Antifungal activity

All isolates were evaluated for their *in vitro* antifungal activity by dual culture assays on NA + potato dextrose agar (1:1) plate against the plant pathogens, *Ustilago scitaminea* and *Ceratocystis paradoxa* according to Singh et al. (2013, 2014). The strains exhibiting more than 50% inhibition in mycelial growth were considered as promising antagonists.

### Nitrogen fixation by acetylene reduction assay (ARA)

Nitrogen-fixing ability of all isolate was tested by using ARA previously described by Hardy et al. (1968). All bacterial isolate was inoculated in a 25 mL flask containing 10 mL semi solid JNFb medium (Table S1) and bacteria were grown at 30 ± 2°C for 3 days. Five Percent air from the tubes was replaced by acetylene through a syringe, incubated for 12 h, 0.5 mL gas was withdrawn from the tube, and ethylene formation was analyzed through a gas chromatograph (GC-17A, Shimadzu, Kyoto, Japan) with a flame ionization detector and a column filled with DB-1701 (Agilent, Santa Clara, USA).

### 1-Aminocyclopropane-1-Carboxylate (ACC) deaminase activity

Screening for ACC deaminase activity of all isolates was done based on their ability to use ACC as a sole nitrogen source, a trait that is consequence of the activity of the enzyme ACC deaminase. All the isolates were grown in 10 mL of LB broth medium incubated at 30 ± 2°C at 120 rpm for 24–36 h. The cells were harvested by centrifugation at 10,000 rpm for 5 min and washed twice with sterile 0.1 M Tris-HCl (pH 7.5). And it was spotted on petri plates of the modified nitrogen free Dworkin and Foster (DF) medium (Jacobson et al., 1994). Plates without ACC were used as negative control and those with ACC (3 mM) or (NH_4_)_2_SO_4_ (0.2% w/v) plates were used as positive control. The plates were incubated at 30 ± 2°C for 3–5 days. The isolates had ability to grow on ACC plates confirmed that it possessed ACC deaminase activity.

Quantitative estimations of ACC deaminase activities of the bacterial isolates were determined for selected isolates according to the procedures described by Honma and Shimomura (1978) with a standard curve of α-ketobutyrate ranging between 0.01 and 1.0 μ mole. The absorbance was measured at 540 nm (Shimadzu UV-1800, Japan). Enzyme activity was expressed as μmol mg^−1^ protein h^−1^.

### Genomic DNA extraction

Extraction of total genomic DNA from all the selected bacterial strains was performed using a DNA isolation kit (CWBIO, Beijing, China). After extraction, the quantity, integrity, and quality of the DNA obtained were checked by 0.8% (wt/vol) agarose gel electrophoresis, followed by staining in ethidium bromide, and visualization under UV light. The extracted DNA was further quantified using a nano-photometer (Pearl, Implen-3780, USA).

### PCR amplification of 16S rRNA gene

To amplify the 16S rRNA gene, polymerase chain reaction (PCR) was performed from the genomic DNA of strains using a universal primer pair for pA-F and pH-R (Table 1). The PCR program for 16S rRNA gene included initial denaturation at 95°C for 5 min, 30 cycles of denaturation at 95°C for 1 min, annealing at 55°C for 1 min, extension at 72°C for 1 min and final extension cycle at 72°C for 5 min. Amplified fragments were checked and purified by using PCR purification kit BioFlux (Hangzhou, China) and then sequenced at Sangon Biotech (Shanghai, China).

**Table 1:** PCR primers used for identification, functional genes amplifications and *nifH* gene expression.

**Gene**	**Primer**	**Sequence (5′ → 3′)**	**Product size**	**References**
16S	pA-F	AGAGTTTGATCCTGGCTCAG	1,300–1,500 bp	Edwards et al., 1989
	pH-R	AAGGAGGTGATCCAGCCGCA		
*NifH*	Pol-F	TGCGAYCC-SAARGCBGACTC	360 bp	Poly et al., 2001
	Pol-R	ATSGCCATCATYTCRCCGGA		
PhCA	PhCA-F	TTGCCAAGCCTCGCTCCAAC	1,150 bp	Raaijmakers et al., 1997
	PhCA-R	CCGCGTTGTTCCTCGTTCAT		
PRN	Prn-F	GGGGCGGGCCGTGGTGATGGA	786 bp	Souza and Raaijmakers, 2003
	Prn-R	YCCCGCSGCCTGYCTGGTCTG		
HCN	HCN-F	ACTGCCAGGGGCGGATGTGC	587 bp	Ramette et al., 2003
	HCN-R	ACGATGTGCTCGGCGTAC		
BOX	BOX A1R	CTACGGCAAGGCGACGCTGACG	50–5,000 bp	Rademaker and de Bruijn, 1997
ERIC	ERIC-1	ATGTAAGCTCCTGGGGATTCAC	50–5,000 bp	
	ERIC-2	AAGTAAGTGACTGGGGTGAGCG		
REP	REP-F	IIIICGICGICATCIGGC	50–5,000 bp	
	REP-R	ICGICTTATCIGGCCTAC		
**Q-RT-PCR PRIMER**				
*NifH*	Pol-F	TGCGAYCC-SAARGCBGACTC		Poly et al., 2001
	Pol-R	ATSGCCATCATYTCRCCGGA		
Glyceraldehyde 3-phosphate dehydrogenase	GAPDH-1	CTCTGCCCCAAGCAAAGATG		Niu et al., 2013
	GAPDH-2	TGTTGTGCAGCTAGCATTG		

### Phylogenetic analysis

To perform molecular phylogenetic analysis and evolutionary relationship analysis, the 16S rRNA gene sequences of the isolated *Pseudomonas* strains were compared with reference strain sequences deposited in the National Center for Biotechnology Information (NCBI) GenBank public database. The sequences were aligned by ClustalW (Saitou and Nei, 1987) and the phylogenetic tree was reconstructed using MEGA software version 7.0 (Kumar et al., 2016) and unweighted pair group method with arithmetic mean (UPGMA) (Sneath and Sokal, 1973) in a Kimura two-parameter model (Tamura et al., 2004). To obtain the confidence values, the gaps were treated by pairwise deletions and bootstrap analysis was carried out by the method of Felsenstein (1985) using 1,000 pseudoreplications.

### Amplification, cloning, and sequencing of the *nifH* gene

Isolated DNA template from all 30 selected strains were used to amplify a conserved region of the *nifH* gene fragment by PCR, according to the method of Poly et al. (2001) using the primers PolF and PolR (Table 1).

### Detection of antibiotic genes

Genomic DNA of *Pseudomonas* strains was used for the PCR amplification of genes involved in the biosynthesis of three different types of antibiotics genes:- phenazine-1-carboxylic acid (PhCA) (Raaijmakers et al., 1997), pyrrolnitrin (PRN) (Souza and Raaijmakers, 2003), and hydrogen cyanide (HCN) (Ramette et al., 2003) respectively (Table 1). The antibiotics PhCA and PRN are broad-spectrum antibiotics produced by several strains of *Pseudomonas*, which play an important role in the suppression of multiple plant pathogenic fungi, whereas HCN is a broad-spectrum antimicrobial compound that play a key role in the biological control of root diseases by many plant-associated *Pseudomonas* isolates.

### Genetic diversity studies

The genetic PCR fingerprinting was carried out using repetitive consensus with BOX-PCR (based on primers targeting the highly conserved repetitive DNA sequences of the BOXA subunit of the BOX element), ERIC-PCR (based on primers targeting the highly conserved enterobacterial repetitive intergenic consensus) and REP-PCR (based on primers targeting the repetitive extragenic palindromic sequence). The genomic fingerprints were obtained as described by Rademaker and de Bruijn (1997) to determine phylogenetic relatedness and sequences of different primers (Table 1). All PCR reactions were carried out in Peltier Thermal Cycler BIORAD. PCR amplifications were performed in a 25 μL reaction volume, the reaction mixture and conditions are given in Table S2. Amplification was analyzed by 1.6% agarose gels electrophoresis containing 0.5 μg mL^−1^ ethidium bromide. A low range ladder (TaKaRa, Dalian, China) was used as molecular size marker. The gels were visualized and gel images were documented in Bio-Rad gel documentation system. The profiles generated by genetic diversity analysis were compared by calculating Jaccard's similarity coefficient for each pairwise comparison and dendrogram was constructed from the similarity matrix by the unweighted pairgroup method with arithmetic average (UPGMA) using NTSYS pc, version 2.02h. All the experiment was carried out in three replicates.

### BIOLOG^(R)^ phenotypic assays

Assays of utilization of potential carbon (C), nitrogen (N), and tolerance to different osmotic and pH conditions were tested using BIOLOG Phenotype Micro-Array™ plates GENIII, PM3B, PM9, and PM10 (Biolog Inc., Hayward, CA). The number of all the possible conditions was assayed in the four different types of microplates. GENIII plates were used to study C sources metabolism, and PM3B plates to assess N metabolism sources, respectively. In addition, PM9 and PM10 plates were used to test the growth under various stress conditions and different pH (Bochner, 2009; Mazur et al., 2013). Two isolates (CY4 and CN11) were grown at 30 ± 2°C on LB agar medium and then suspended in an inoculation fluid (IF) after washing to get the transmittance of 90–98% according to procedure. A 100 mL cell suspension was then transferred into the 96 wells of all Micro-Plate, and then incubated at 30 ± 2°C for 48 h to allow the phenotypic fingerprint to form. During incubation there is an increased respiration in the wells and cells can utilize different sources and grow. Increased the respiration causes reduction of the tetrazolium dye and forming a purple color. After incubation the readings were obtained using automated BIOLOG^(R)^ Micro-Station Reader according to the instructions of the manufacturer.

### Green fluorescent protein technique

#### Plasmid transformation

The *Pseudomonas* strains (CY4 and CN11) resistant to ampicillin (40 μg mL^−1^) were chosen as recipients for genetic tagging with GFP-pPROBE-pTet^r^-OT. Both the strains were sensitive to kanamycin (100 μg mL^−1^). Plasmid pPROBE-pTet^r^-OT containing the GFP and kanamycin genes expressed under the control of a Tet^r^ promoter was introduced by biparental mating using donor strain *E. coli* TG1. Plasmid pPROBE-pTet^r^-OT is a derivative of plasmid pBBR1, which is a small (2.6 kb), broad-host range plasmid and stably maintained in a number of gram (+) and (−) bacteria. The recipient strains and donor strains were mixed at a ratio of 1:2. An aliquot (100 μL) of this mixture was spread onto LB agar. After overnight incubation of the plates at 30°C, the bacteria were washed off the plates and suitable dilutions of the cultures were plated onto selective media containing ampicillin (40 μg mL^−1^) and kanamycin (100 μg mL^−1^). Ex-conjugants showing green fluorescence under UV illumination were selected for further study.

#### Inoculation of micro-propagated sugarcane plantlets

Sugarcane micro-propagated plantlets were inoculated with *Pseudomonas* strains and GFP-tagged *Pseudomonas* strain/pPROBEpTet^r^-TT as described by Oliveira et al. (2002). Five individual rooted plantlets were transferred into a glass bottle of 300 mL capacity containing 50 mL of liquid one-tenth MS medium (sucrose and basal salt mixture) (Reis et al., 1999). Two days after plant transfer, the media free of clear microbial contamination were inoculated with bacterial suspension, providing an initial bacterial cell numbers of approximately 2.0 × 10^5^ mL^−1^ medium. Plantlets without inoculation were prepared as control. Plantlets were grown in a growth chamber at 30°C with a 14 h photoperiod at a 60 μ moL m^−2^ s^−1^ photon flux density.

#### Laser scanning confocal microscopy (olympus SXZ16)

Three days after inoculation, sugarcane tissue culture plantlets (inoculated and un-inoculated) were taken out from the tubes and sugarcane plantlets were washed with autoclaved distilled water. After cut into small pieces, the root and stem tissues were mounted on bridge slide with 10% (v/v) glycerol. Whole root parts and optical sections of the root and stem pieces were observed with a Leica DMI 6000 microscope attached to a Leica TCS SP5 laser scanning confocal microscope (Leica Microsystems, Mannheim, Germany). GFP fluorescence was detected with an emission band from 500 nm to 530 nm while root auto fluorescence was detected by adjusting the band width from 600 nm to 800 nm depending on the intensity of auto fluorescence (Lin et al., 2012).

### RNA extraction and cDNA synthesis

Total RNA was extracted using Trizol reagent (Tiangen, Beijing, China), according to the manufacturer's instructions. DNA contamination of the RNA was removed by DNase I (Promega, USA). The extracted RNA was further quantified using a Nano photometer (Pearl, Implen-3780, USA). Synthesis of first-strand cDNA from the RNA was carried out using the Prime-ScriptTM RT Reagent Kit (TaKaRa, Dalian, China).

### Quantitative real-time polymerase chain reaction

Expression patterns of the target *nifH* gene during plant-microbe interaction were studied in a greenhouse experiment for the selected strains (CY4 and CN11) in two sugarcane varieties (GT11 and GXB9) at 90 and 120 days, as well as for a control. Leaf samples of both sugarcane varieties were used as the experimental material. The relative expression of the target genes was calculated as the expression level of the inoculated sample minus the level of the control at each corresponding time point. Each qRT-PCR experiment was conducted in triplicate. qRT-PCR was carried out with SYBR Premix Ex Tap™ II (TaKaRa, Japan) in Real Time PCR Detection System (Bio-Rad, USA). Reactions were carried out in a final volume of 20 μL, which contained 10 μL SYBR Premix Ex Tap™ II, 2 μL template (10 × diluted cDNA), 0.8 μL of each 10 μM primer and 6.4 μL ddH_2_O. PCR with distilled water as the template was performed as a control. The primer sequences for *nifH* are presented in Table 1. The qRT-PCR program was as follows: 95°C for 30 s, followed by 40 cycles of 95°C for 5 s and 60°C for 20 s (Niu et al., 2013). Melting curve analysis was conducted at the end of amplification to confirm the specificity of the reaction. The 2^−ΔΔCt^ method was used to quantify the relative gene expression (Livak and Schmittgen, 2001).

### Statistical analysis

Experimental data was analyzed using standard analysis of variance (ANOVA) followed by Duncan's multiple range test (DMRT). Standard errors were calculated for all mean values. Differences were considered significant at the *p* ≤ 0.05 level. All biochemical experiments were performed in triplicate, and the results were expressed as mean values. OrigiPro 9.1 (2013) software was used for the principle component analysis (PCA).

## Results

### Analysis of chemical and trace elements composition in the soil

The soil texture was analyzed, and characterized as a medium loam. The pH varied from a low of 5.99 to a high of 6.70, and the electrical conductivity of the soil samples varied from 111.0 to 72.8 μS cm^−1^. The chemical analysis of the soil revealed that the total amounts of nitrogen (N), phosphorus (P) and potassium (K) were 0.30, 0.43, and 13.81 g kg^−1^, respectively, at the time of sampling, (April 2015); calcium and magnesium levels were 784.6 and 147.6 mg kg^−1^, respectively. The amounts of trace elements (mg kg^−1^) were Fe, 106.14; Mn, 85.22; Zn, 7.49; B, 0.42; ${\text{SO}}_{4}^{2-}$, 136.67; Cl^−^, 30.64.

### Isolation and PGP potential

A total of 350 bacterial strains were isolated from the sugarcane rhizospheric soil samples. Of these, 100 strains were selected on basis of morphology. These selected strains were further tested by *in vitro* screening for antifungal activity against sugarcane pathogens (*U. scitaminea* and *C. paradoxa*), and for selected PGP traits, as well as for nitrogenase activity. PGP activities were evident from the ability of the selected isolates to produce plant hormones. Of these, only 30 strains were selected that showed various different PGP traits, such as P-solubilization, or siderophore, ammonia, HCN, ACC, or indole-3-acetic acid IAA production, or acetylene reduction activity for molecular identification. In the case of phosphate solubilization, out of the 30 isolates, 26 (87%) isolates showed halo zone formation on Pikovskaya's agar plates, confirming their ability that solubilized the tricalcium phosphate in the medium. An *in vitro* siderophore production assay revealed that 20 (66.67%) of the strains were able to produce siderophores, 40% of the strains were able to produce ammonia, and 43.33% were able to produce HCN. In the case of qualitative screening of ACC-utilizing bacterial strains, only 18 (60%) exhibited ACC deaminase activity, the strains consumed 3 mM ACC in DF-ACC medium after 48 h incubation at 30 ± 2°C and the color in the DF-ACC medium containing the bacterial strains appeared weaker compared with the non-inoculated medium. In the quantitative estimation of ACC deaminase activity, all strains ranged from 77.0 to 15.13 μmoL mg^−1^ h^−1^, with a maximum activity in CY4 and a minimum in the BA2 strain (**Table 3**). Twenty-one isolates showed antagonistic activity against sugarcane pathogens. Fourteen isolates were antagonistic to *U. scitaminea* (46.66%), and eleven were antagonistic to *C. paradoxa* (36.67%) (Table 2).

**Table 2:** *In vitro* biochemical characterization of antagonistic *Pseudomonas* species on the plant growth promotion traits isolated from sugarcane.

**Culture code**	**Phosphate**	**Siderophore**	**Ammonia**	**HCN**	**ACC**	**Antifungal activity**	
						***Ustilago scitaminea***	***Ceratocystis paradoxa***
CoY1	++	+++	−	−	+	++	−
CoA5	++	−	++	−	−	+	++
CoA6	++	+++	++	−	−	+	++
AY1	++	++	−	−	−	++	−
AY2	+++	+++	−	−	+	−	−
AA8	++	−	−	+++	−	++	+
AN15	−	+++	++	++	−	−	+++
BY2	++	+++	−	++	+	++	−
BY3	+++	−	−	−	−	++	−
BY10	+++	−	−	−	+	−	++
BA2	++	−	−	−	+	++	−
BA4	+++	−	−	−	−	++	−
BA7	+++	−	+++	−	−	−	−
BA12	+++	+++	−	++	+	++	−
BA13	+++	−	+++	−	−	−	+
BA17	++	+++	−	++	+	−	−
BN6	++	+++	+++	+++	−	−	−
BN7	++	+++	+++	+++	−	−	−
CY4	++	++	+++	++	+	+	−
CY7	++	++	−	−	+	+	++
CA5	++	+++	+++	+++	+	−	−
CA7	+++	+++	−	+++	+	−	+++
CN1	+++	++	−	−	+	−	−
CN2	−	++	+++	−	+	−	−
CN3	+++	−	−	−	+	+	−
CN9	+++	+++	−	++	−	−	+
CN11	+++	+++	+++	+++	+	−	+
CN12	+++	−	−	−	+	−	+++
CN15	−	+++	−	+++	+	−	−
CN20	−	++	++	−	+	+	−

*+, showing low activity; ++, moderate activity; +++, strong activity; −, showing no activity*.

*Phosphate and Siderophore activity of the bacterial isolates 3 mm or greater clear zone of inhibitions on suitable medium after 3–5 days of incubation at 30 ± 2°C*.

*Antifungal activity by dual culture plate measured as zone of inhibition after 3–5 days of incubation at 26 ± 2°C*.

Biosynthesis of IAA showed differences among the strains, which are summarized in Table 3. The quantitative production of IAA varied from 312.07 to 13.12 μg mL^−1^ in tryptophan supplemented medium, and a higher level of production was recorded in strain AN15, and a lower one in CN20. In the case of medium without tryptophan, the maximum IAA production was observed from the strain BN7 (23.24 μg mL^−1^) and the minimum from CoA5 (12.92 μg mL^−1^). Nitrogen fixation efficiency of all strains was estimated by the acetylene reduction assay (ARA) under laboratory conditions. The data revealed considerable variability in the nitrogenase activity among the studied strains, which ranged from 108.30 to 6.16 μmoL C_2_H_2_ h^−1^ mL^−1^. On average, under our experimental conditions, the strain CoA6 fixed higher amounts and AY1 fixed lower amounts of nitrogen as compared to the other strains (Table 3).

**Table 3:** Screening of different *Pseudomonas* species for IAA, ARA, and ACC deaminase activity.

**Culture Code**	**IAA (μg mL^−1^)**		**ARA (μmoL C_2_H_2_h^−1^ mL^−1^)**	**ACC (μmoL mg^−1^ h^−1^)**
	**A-Tryptophane**	**P-Tryptophane**		
CoY1	21.84^a^	118.12^e^	13.61^ijk^	30.32^ghi^
CoA5	12.92^k^	130.86^c^	18.41^g^	–
CoA6	18.92^bc^	153.31^b^	108.30^a^	–
AY1	15.27^fgh^	76.19^f^	6.16^n^	–
AY2	14.23^g−k^	128.35^d^	17.46^gh^	44.23^d^
AA8	14.23^g−k^	20.63^h^	22.32^ef^	–
AN15	13.90^g−k^	312.07^a^	25.91^bcd^	–
BY2	17.44^cde^	17.43^ij^	22.34^ef^	32.92^fgh^
BY3	16.47^ef^	16.32^ijk^	25.71^b−e^	–
BY10	13.84^g−k^	14.23^kl^	29.26^b^	19.04^k^
BA2	16.38^ef^	16.37^ijk^	19.30^fg^	15.13^k^
BA4	17.17^de^	17.40^ij^	27.08^bcd^	–
BA7	13.92^g−k^	13.74^kl^	25.59^cde^	–
BA12	15.38^fg^	15.37^jkl^	26.04^bcd^	36.49^ef^
BA13	14.72^g−j^	14.92^jkl^	18.12^g^	–
BA17	15.14^fgh^	15.08^jkl^	23.83^de^	60.78^b^
BN6	15.21^fgh^	15.21^jkl^	17.34^gh^	–
BN7	23.13^a^	23.12^g^	26.08^bcd^	–
CY4	20.25^b^	310.63^a^	26.33^bcd^	77.00^a^
CY7	14.90^f−i^	14.90^jkl^	28.30^bc^	24.33^j^
CA5	13.84^g−k^	13.84^kl^	8.49^lmn^	25.97^ij^
CA7	14.51^g−k^	14.36^kl^	10.82^kl^	52.92^c^
CN1	13.62^h−k^	13.59^l^	6.87^mn^	32.72^fgh^
CN2	13.32^ijk^	13.26^l^	13.62^ijk^	29.68^hi^
CN3	13.99^g−k^	14.06^kl^	18.41^g^	34.99^fg^
CN9	23.24^a^	23.31^g^	14.24^hij^	–
CN11	23.00^a^	23.26^g^	16.89^ghi^	33.17^fgh^
CN12	18.16^cd^	18.29^i^	11.11^jkl^	29.64^hi^
CN15	13.77^g−k^	13.77^kl^	9.71^lm^	40.23^de^
CN20	13.12^jk^	13.12^l^	18.58^g^	15.62^k^
SEM	0.499	0.785	1.082	1.479
CD (*P* = 0.05)	1.413	2.224	3.063	4.252
CV (%)	5.3	2.5	8.6	7.3

*Means followed by same letter within a row are not significantly different (P ≤ 0.05) according to Duncan's Multiple Range Test (DMRT). SEM standard error of the difference between means, CD, critical difference; CV, coefficient of variation*.

### Molecular identification and phylogenetic analysis

**Figure 1:**
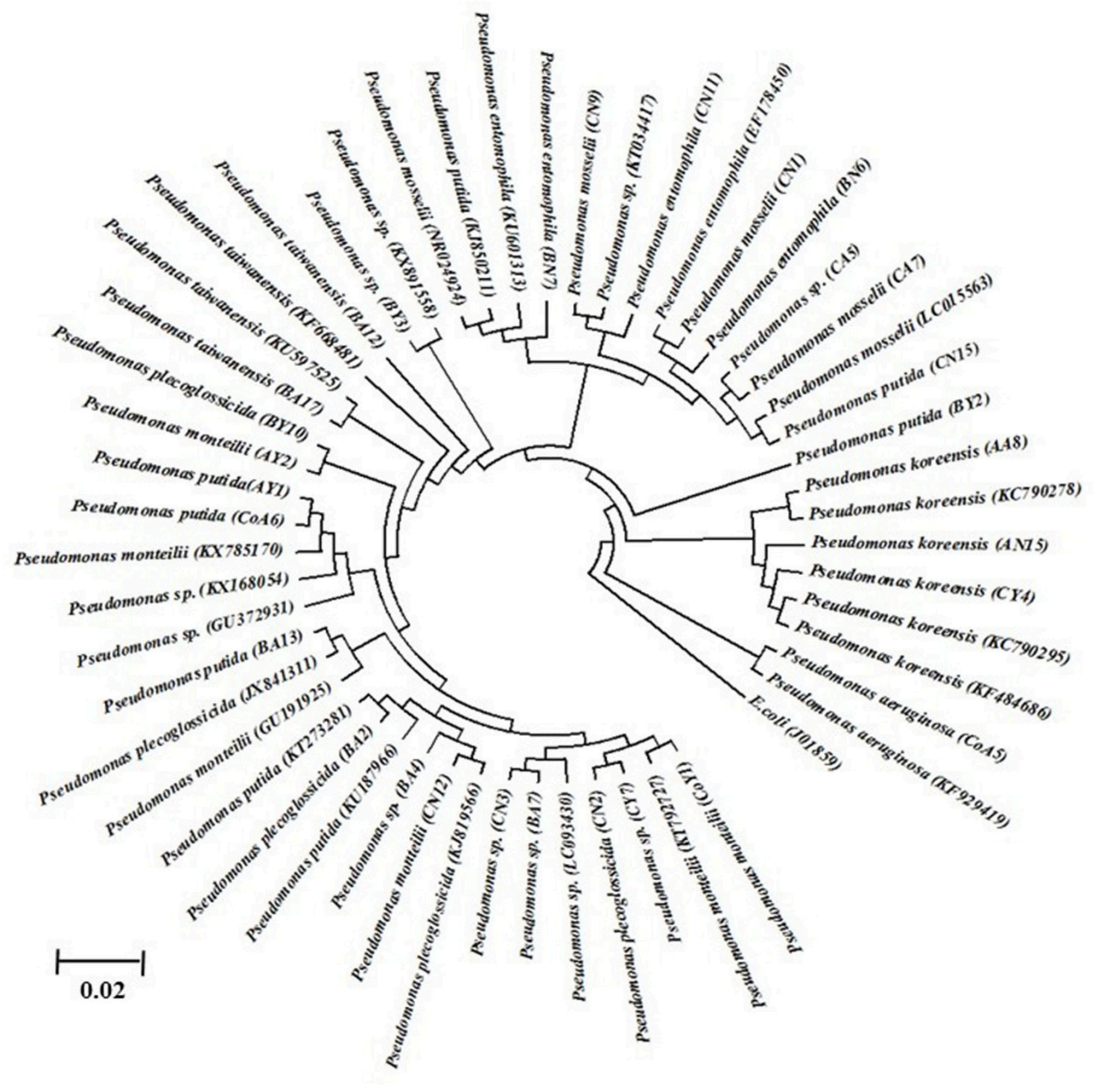
The 16S rDNA phylogenetic tree of *Pseudomonas* isolates from sugarcane. The evolutionary history was inferred using the UPGMA method. Bootstrap values of 1,000 replications are indicated as percent confidence values for particular branching. All positions containing gaps and missing data were eliminated. Sequences indicated in code were determined in this study. The *E. coli* was used as an out group.

Identification of all isolates was performed based on partial 16S rRNA gene sequencing. The strain sequences obtained were compared using the BlastN tool, with NCBI GenBank database nucleotide sequences and similarity values ≥97% being obtained. The results are suggestive that all the isolates belonged to different species of *Pseudomonas*, i.e., *P. monteilii* (3), *P. aeruginosa* (1), *P. putida* (5), *P. koreensis* (3), *P*. spp. (7), *P. plecoglossicida* (3), *P. taiwanensis* (2), *P. entomophilla* (3), and *P. mosselii* (3), and the nucleotide sequence data have been submitted to the NCBI GenBank database (Table 4). These DNA sequences were aligned and used to reconstruct a phylogenetic tree, possessing five clusters with 1,000 bootstrap samplings with representative strains of related taxa, as shown in Figure 1.

**Table 4:** Identification of putative plant growth-promoting bacterial strains isolated from sugarcane based on 16S rDNA sequence and NCBI GenBank databases.

**Isolates**	**16S rRNA gene**	**% Similarity**	**Accession number match**	**No. of nucleotides**	**Accession numbers**
CoY1	*Pseudomonas monteilii*	97	KX785170	1,501	KY460972
CoA5	*Pseudomonas aeruginosa*	99	KF929419	1,438	KY460973
CoA6	*Pseudomonas putida*	99	KC952984	1,440	KY460974
AY1	*Pseudomonas putida*	99	KU187966	1,414	KY460975
AY2	*Pseudomonas monteilii*	99	LC015566	1,383	KY460976
AA8	*Pseudomonas koreensis*	99	KC790278	1,424	KY460977
AN15	*Pseudomonas koreensis*	99	KC790275	1,446	KY460978
BY2	*Pseudomonas putida*	98	KT759148	1,040	KY460979
BY3	*Pseudomonas* sp.	99	KX891558	1,162	KY460980
BY10	*Pseudomonas plecoglossicida*	99	LT671913	1,396	KY460981
BA2	*Pseudomonas plecoglossicida*	97	JQ976892	1,436	KY460982
BA4	*Pseudomonas* sp.	99	GU372931	1,433	KY460983
BA7	*Pseudomonas* sp.	99	LC093430	1,486	KY460984
BA12	*Pseudomonas taiwanensis*	99	KU597525	1,417	KY460985
BA13	*Pseudomonas putida*	99	KT273281	1,458	KY460986
BA17	*Pseudomonas taiwanensis*	99	KU597525	1,433	KY460987
BN6	*Pseudomonas entomophila*	99	KU601313	1,386	KY460988
BN7	*Pseudomonas entomophila*	99	KU601313	1,409	KY460989
CY4	*Pseudomonas koreensis*	99	KF484686	1,444	KY460990
CY7	*Pseudomonas* sp.	99	GU372931	1,429	KY460991
CA5	*Pseudomonas* sp.	98	KP126777	1,445	KY460992
CA7	*Pseudomonas mosselii*	98	KU550148	1,353	KY460993
CN1	*Pseudomonas mosselii*	99	LC015563	1,419	KY460994
CN2	*Pseudomonas plecoglossicida*	99	KY006168	1,404	KY460995
CN3	*Pseudomonas* sp.	99	KX168054	1,460	KY460996
CN9	*Pseudomonas mosselii*	98	LN995691	1,490	KY460997
CN11	*Pseudomonas entomophila*	98	KU601313	1,398	KY460998
CN12	*Pseudomonas monteilii*	98	KX785170	1,514	KY460999
CN15	*Pseudomonas putida*	98	KJ850211	1,410	KY460100
CN20	*Pseudomonas* sp.	97	KT034417	1,448	KY460101

### Detection of *nifH* and antibiotic genes

**Figure 2:**
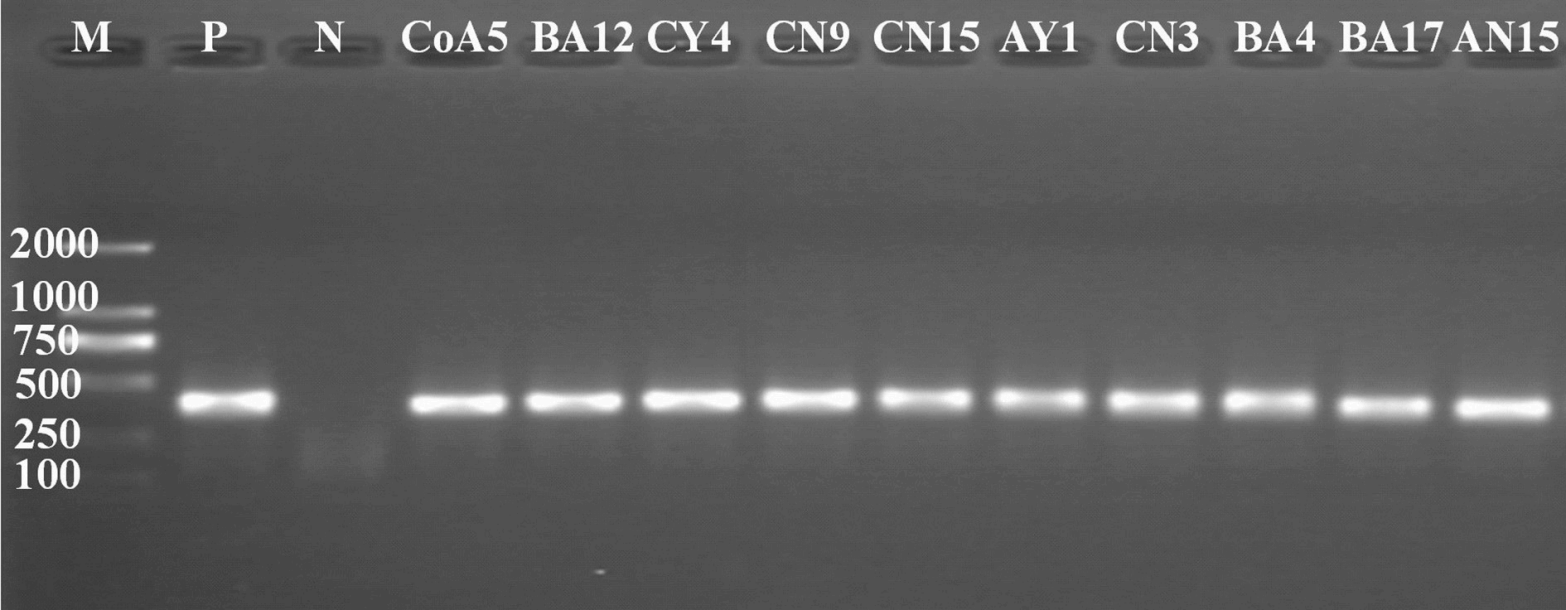
PCR-amplification of *nifH* gene from genomic DNA of *Pseudomanas* species. M, molecular size marker from 100 to 2,000 bp. P is a positive control (*Klebsiella verticola*- DX120E) and N is a negative control (Water). M, molecular size marker (100 bp–2 kb), (Takara).

PCR products of the correct size were amplified from the total genomic DNA extracted from all strains. Of these, 10 strains were positive for *nifH* gene amplification, producing an amplified fragment of about 360 bp (Figure 2); these strains were CoA5, BA12, CY4, CN9, CN15, AY1, CN3, BA4, BA17, and AN15. The All positive strains were selected and used to establish *nifH* clone libraries. A total of ten clones selected from each strain were analyzed by sequencing. All clones were sequenced, analyzed, and identified by BlastN. All sequences were related to the partial *nifH* gene. The clones obtained had similarity levels that varied from 90 to 100% and the NCBI GenBank accession numbers are KY508382–KY508391.

Amplifications of three genes of antibiotic biosynthesis; (involved in biocontrol activity in the biosynthesis of PhCA, PRN, and HCN) were used in this study. Only four strains showed the PhCA related amplified band size of 1.15 kb, and five showed PRN related gene amplification at around 786 bp. In the case of the HCN related gene, nine strains showed positive amplification at 587 bp. All the amplified products were purified and sequenced. The antibiotic related gene sequences were analyzed and identified using the BlastN program, but only the HCN related sequences submitted to the NCBI GenBank database, under accession numbers KY508373–KY508381. On the basis of the above results, we selected two strains (CY4 and CN11) for the study of substrate richness using different Biolog plates (carbon, nitrogen, and osmolytes), and for study of the plant-microbe interaction mechanism by means of GFP.

### Genotypic diversity

**Figure 3:**
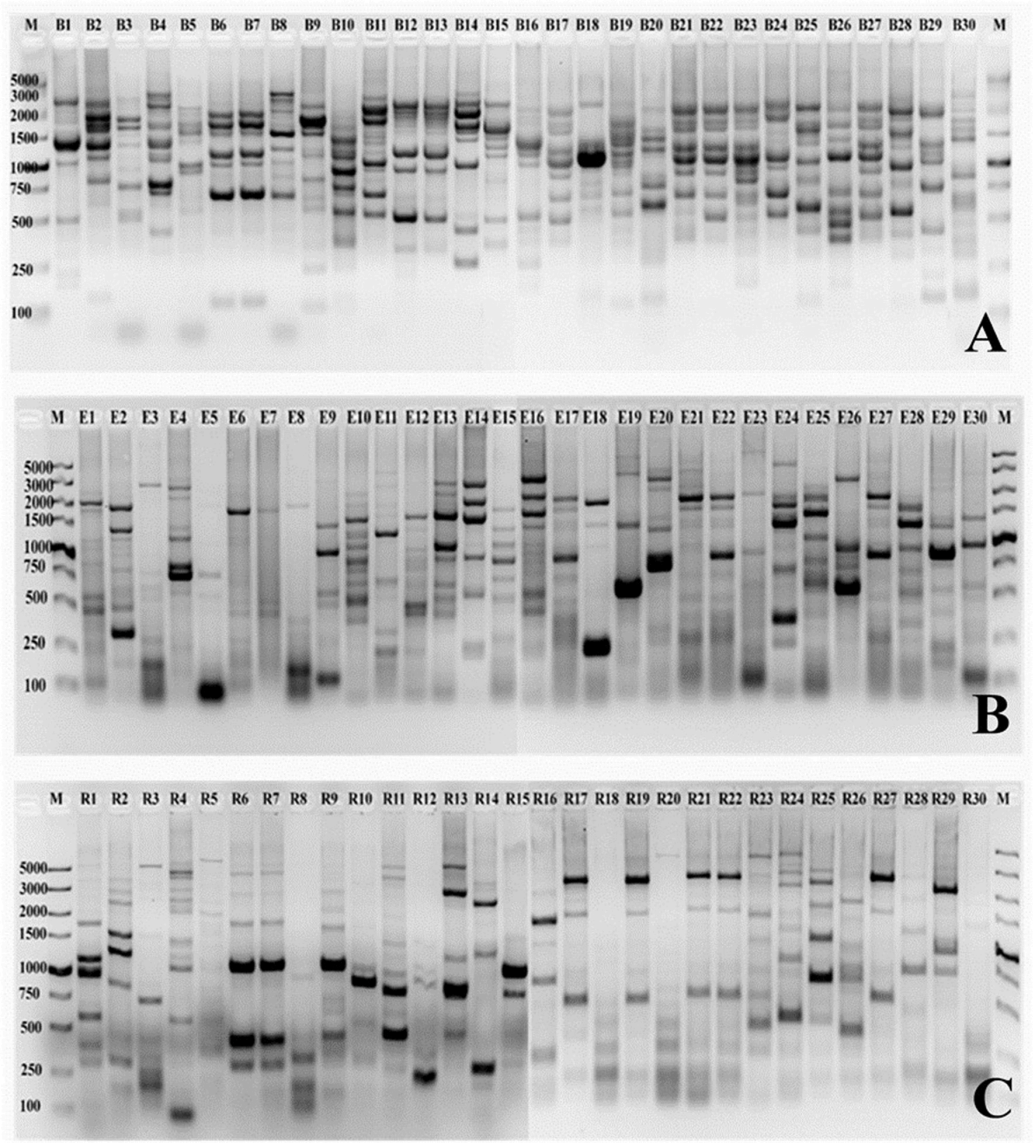
PCR finger printing patterns from genomic DNA of *Pseudomonas* strains isolated from sugarcane. The BOX, ERIC and REP patterns are shown in **(A–C)**, respectively. B1-B30 (BOX), E1-E30 (ERIC), and R1-R30 (REP) is a strain codes used in this study. A 12–15 μL of each product is loaded onto a 1.5% agarose gel. M, molecular size marker (100 bp–5 kb), low range DNA ruler (Takara). Strain codes: 1. CoY1, 2. CoA5, 3. CoA6, 4. AY1, 5. AY2, 6. AA8, 7. AN15, 8. BY2, 9. BY3, 10. BY10, 11. BA2, 12. BA4, 13. BA7, 14. BA12, 15. BA13, 16. BA17, 17. BN6, 18. BN7, 19. CY4, 20. CY7, 21. CA5, 22. CA7, 23. CN1, 24. CN2, 25. CN3, 26. CN9, 27. CN11, 28. CN12, 29. CN15, and 30. CN20.

In the present study, the genetic diversity fingerprints of all the selected strains isolated from sugarcane rhizosphere were investigated through BOX, ERIC, and REP-PCR fingerprints. A number of polymorphic bands ranging between 50 bp and about 5 kb were observed, and the DNA fingerprints were clearly differentiated from each other. Nearly all the selected strains showed high-quality DNA fingerprint profiles generated with each primer set (Figure 3). The fingerprint patterns of the thirty isolates generated by BOX, ERIC, and REP-PCR were complex, producing a large number of polymorphic bands of variable intensity. Differences among strains were assessed visually, on the basis of the banding patterns of PCR products. BOX PCR generated a total of 268 bands ranging from 150 bp to 4 kb for all the 30 selected *Pseudomonas* strains studied. The maximum numbers of bands (12) were observed for CN1 and CN20 while the lowest (5) was found in the case of BN7 (Figure 3A). A total of 229 bands were identified by ERIC-PCR for all the *Pseudomonas* strains, and the band sizes ranged from 50 bp to 4 kb. CoY1, CoA5, BY10, BA17, CA5, and CN15 showed the highest number of bands (11), while AN15 showed the lowest number of bands (3) (Figure 3B). For REP-PCR, 210 bands were identified, of approximately 50 bp to 5 kb, and faint bands were also frequently observed. The highest number of bands (12) was observed for AY1, while the lowest (4) was found in the case of BN7 and CN11 (Figure 3C). In the case of all these PCRs, BOX-PCR fingerprints revealed a high genotypic diversity for all the *Pseudomonas* strains.

**Figure 4:**
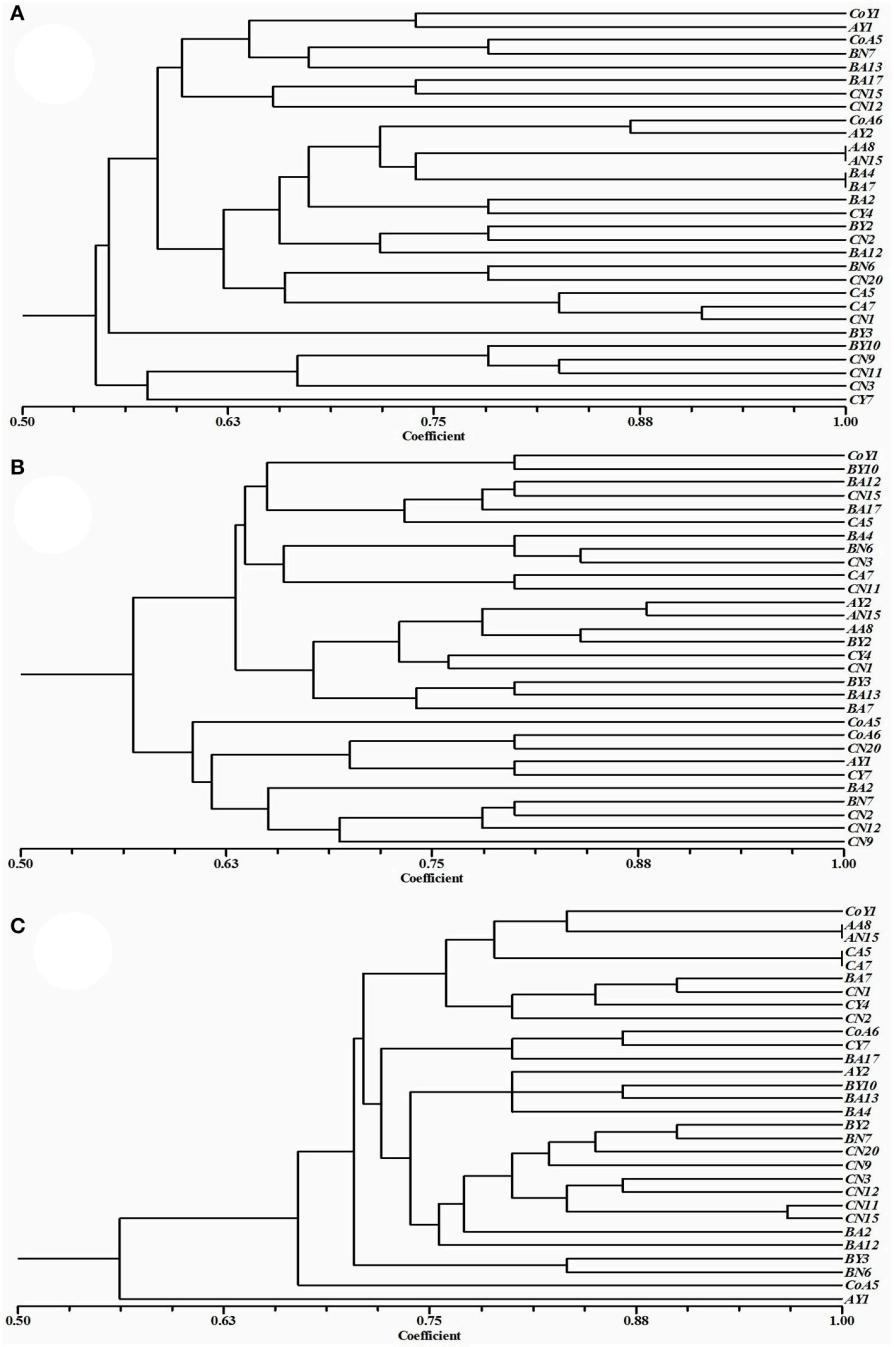
Cluster analyses of **(A)** BOX, **(B)** ERIC, and **(C)** REP-PCR fingerprints showing the genotypic diversity of *Pseudomonas* species isolated from sugarcane. Dendrogram was obtained from the similarity coefficient calculations and clustering was done using unweighted pair-grouping method based on arithmetic averages (UPGMA) using NTSYS software and the Jaccard coefficient.

To determine the relatedness of the isolates, a dendrogram was reconstructed based on BOX, ERIC, and REP-PCR fingerprint bands (Figure 4), and the data were analyzed by using Jaccard similarity coefficients and the neighbor-joining clustering method based on pair-wise similarity coefficients with UPGMA. The dendrogram generated through BOX PCR fingerprints revealed two major clusters, one containing five strains and the other containing 25 strains (Figure 4A). The clustering was clear, and represented 28 distinct clusters of *Pseudomonas* spp. In the ERIC-PCR, all 30 *Pseudomonas* spp. showed two major clusters containing 20 and 10 strains (Figure 4B), all grouped into different clusters. As with BOX and ERIC-PCR, the dendrogram generated based on the REP-PCR fingerprints also showed two major clusters, containing one and 29 strains (Figure 4C) respectively, and all 30 strains were grouped into 28 different clusters of *Pseudomonas* spp.

### Substrate utilization patterns

**Figure 5:**
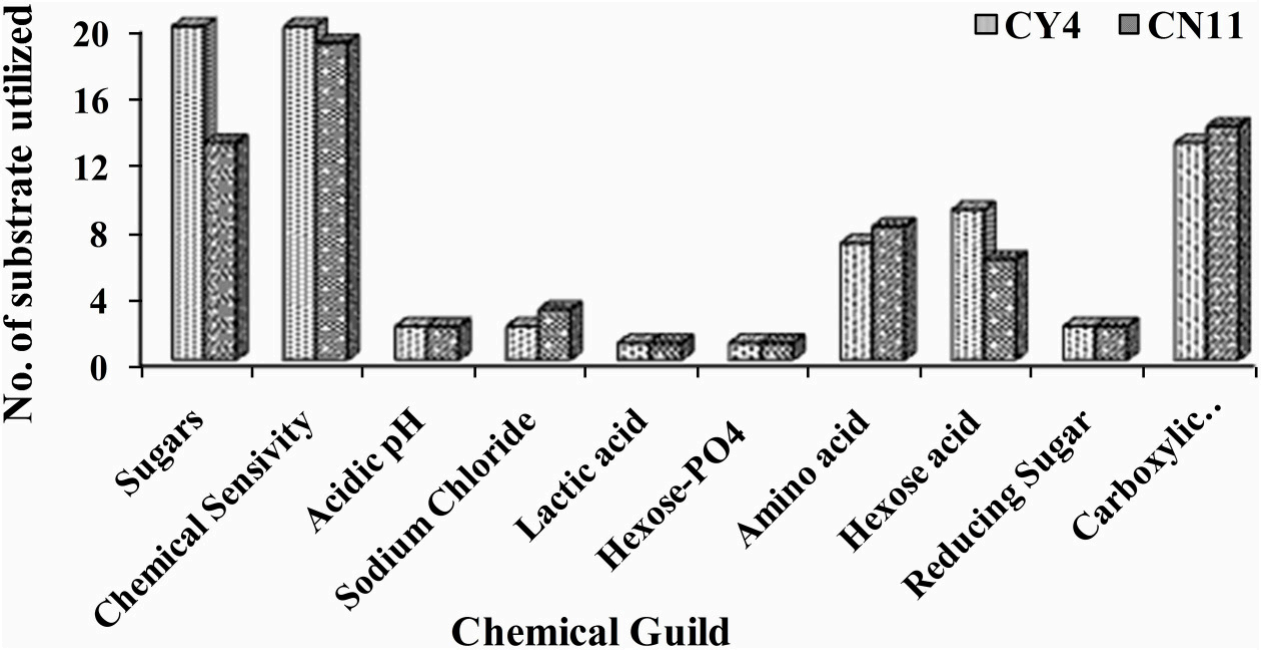
Metabolic differences among CY4 and CN11 in presence of sugars, chemical sensivity, acidic pH, sodium chloride, lactic acid, hexose PO_4_, amino acid, hexose acid, reducing sugar, and carboxylic acid using BIOLOG Phenotype Micro-Array™ plates GNIII.

Selected strains were tested for metabolic potential with several compounds as sole sources for carbon (C), using GNIII and nitrogen (N) PM3B micro-plates. Strain tolerance of osmotic stress was examined with PM9 micro-plates, and metabolic activity over a wide range of pH, 3.5–10, was determined by using PM10 micro-plates. The Biolog system was used to detect the biochemical, physiological, and chemical sensitivity of strains on the basis of substrate richness (Table S3). The results indicated that strain CY4, followed by CN11, showed the highest utilization of carbon sources, i.e., sugars (28.17%), carboxylic acids (18.30%), hexose acids (12.68%), and amino acids (9.86%), and the highest chemical sensitivity (86.95%) (Figure 5).

**Figure 6:**
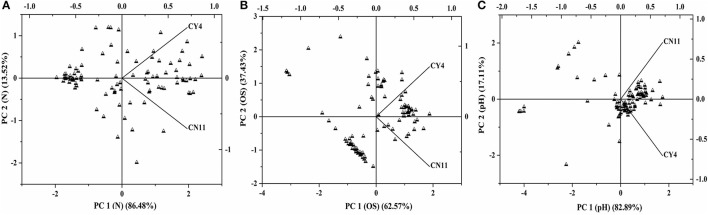
The scatterplots of selected two strains *Pseudomonas koreensis* (CY4) and *Pseudomonas entomophila* (CN11) were analyzed through principle component analysis (PCA) under the different treatments by using the BIOLOG^(R)^ micro-plates **(A)** nitrogen, **(B)** osmolytes, and **(C)** pH.

A qualitative analysis of metabolic differentiation between the selected strains was performed through principal component analysis (PCA), using PM3B, PM9, and PM10 (Figure 6 and Table S4). The main principle for the grouping of every substrate group to the individual component was the relationship between different metabolites and strain utilization. For these studies, separate evaluations of nitrogen, osmolytes, and pH were performed. A scatter plots diagram of PCA showed the nitrogen of different components accounted for 86.48% of the metabolic variation in PC1 for the selected strains (Figure 6A). The osmolytes accounted for 62.57% of the variance and the pH for 82.89% of the variance observed in PC1 (Figures 6B,C). Based on the data obtained through the Biolog analysis, the diversity index was measured and was evident for the selected strains, which showed high metabolic diversity (Table 5).

**Table 5:** Substrate richness diversity indices calculated for nitrogen, osmolytes and pH through Biolog for selected strains *Pseudomonas koreensis* (CY4) and *Pseudomonas entomophila* (CN11).

**Index measure**	**Diversity indices through Biolog Micro-Plates**					
	**Strain CY4**			**Strain CN11**		
	**Nitrogen**	**Osmolytes**	**pH**	**Nitrogen**	**Osmolytes**	**pH**
	**PM3B**	**PM9**	**PM10**	**PM3B**	**PM9**	**PM10**
Dominance D	0.01507	0.01634	0.01178	0.01488	0.01137	0.01131
Simpson 1-D	0.9849	0.9837	0.9882	0.9851	0.9886	0.9887
Shannon H	4.327	4.193	4.46	4.33	4.499	4.501
Evenness e^∧^H/S	0.7891	0.6899	0.9006	0.7912	0.9368	0.9385
Brillouin	1.357	2.177	2.729	1.311	2.072	2.522
Menhinick	10.71	8.977	7.258	11.29	7.911	7.339
Margalef	25.58	21.56	19.31	26.51	20.86	19.64
Equitability J	0.9481	0.9187	0.9771	0.9487	0.9857	0.9861
Fisher alpha	0	282	87.25	0	119.6	90.32
Berger Parker	0.01244	0.01749	0.01143	0.01384	0.01358	0.01169

### Colonization of sugarcane plants by GFP-tagged bacteria

**Figure 7:**
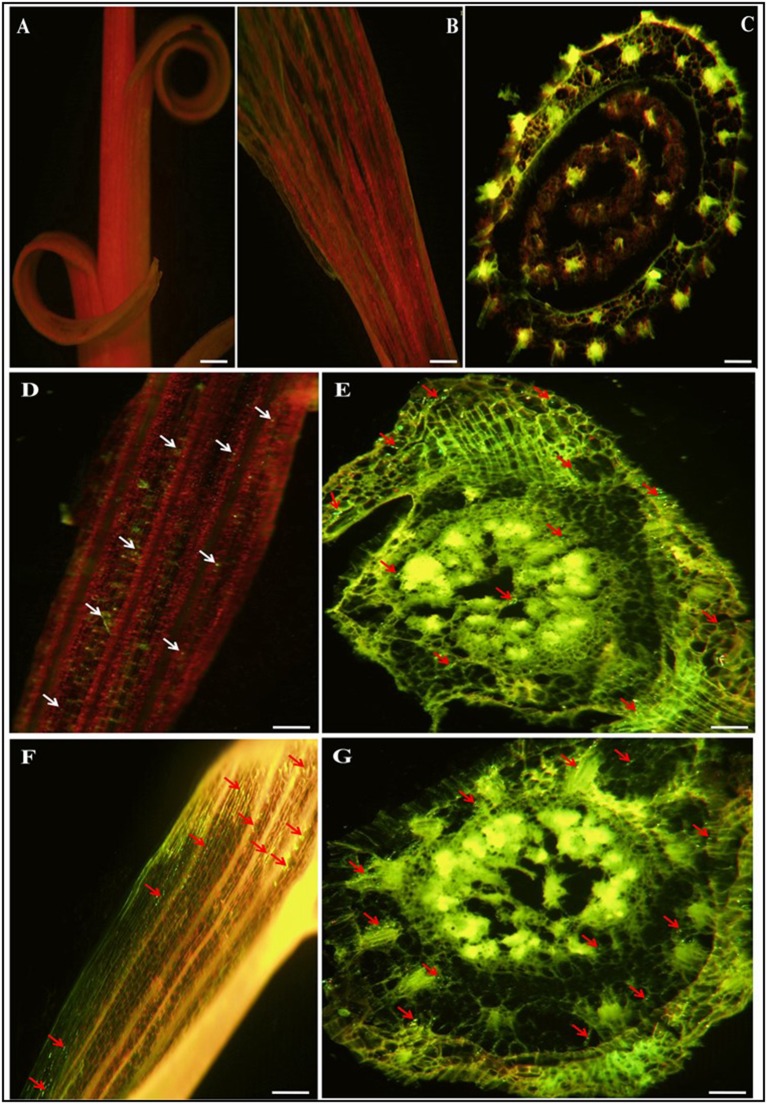
Confocal laser scanning micrographs images showing gfp-tagged strains (CY4 and CN11) colonized in and on roots and leaves of sugarcane micropropagated plantlets GT11 (variety). **(A–C)** is control sugarcane plantlets parts i.e., stem, leaf and root, without inoculated strains. Confocal microscopic images **(D–G)** present inoculated bacterial GFP fluorescence (500–530 nm) in green dots and auto-fluorescence in everywhere in leaf and root. Arrow heads point indicates bacterial cells present in a single or grouped of bacteria. **(D,E)** represents CY4 and **(F,G)** is CN11 strain. Bars present 50 μm.

Visualization of root colonization was carried out by CLSEM. This technique facilitated study of the interaction of the selected potential strains in sugarcane. Bacterial colonization in the internal tissues of plants was observed in almost all parts of the sugarcane plant. After 72 h of incubation with the inoculated strains (CY4 and CN11), it was observed that the bacterial cell density had increased, and green fluorescent cells were distributed throughout the plant organs, including roots, stems, and leaves (Figure 7). No fluorescent bacteria were detected by CLSEM in the uninoculated plants served as a control (Figures 7A–C). In roots, the GFP-tagged bacteria were observed by CLSEM to colonize mostly mature root hair zones. Many of the cells were over shadowed by the green fluorescence light emitted from the cell walls of the epidermal cells, endodermal, xylem vessels, and junction sites between the primary and lateral root zones, which were excited by the blue light of the fluorescence microscope (Figures 7E,G). In the case of leaves, colonization of GFP-tagged bacteria was clearly observed through the green auto-fluorescence emitted as small dots in all plant parts (Figures 7D,F), in the case of roots, the cells emitted fluorescence in the maturation zone and within the body of the root, detected by CLSEM in transvers hand-cut optical sections.

### *nifH* gene expression determined by qRT-PCR

**Figure 8:**
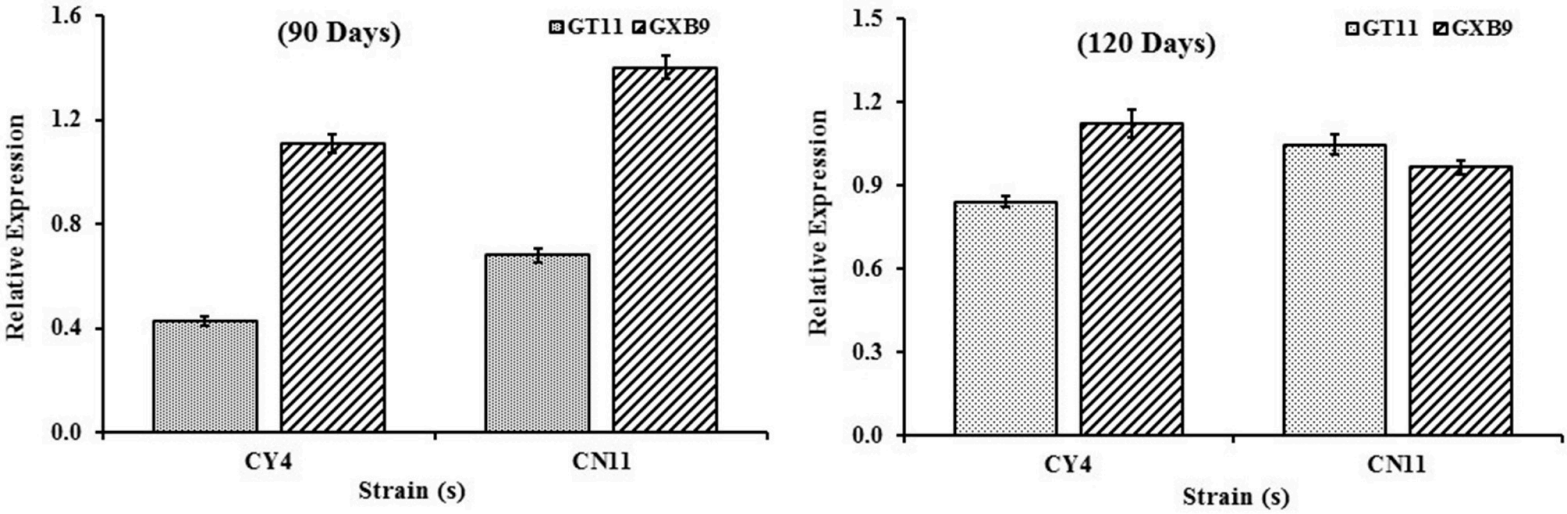
qRT-PCR analysis of the *nifH* expression patterns in sugarcane varieties (GT11 and GXB9) and potent strains (*Pseudomonas koreensis*-CY4 and *Pseudomonas entomophila*-CN11) interaction. Data were normalized to the GAPDH expression level. All data points (with the deduction of their controls) are the means ± SE (*n* = 3).

The *nifH* gene expression pattern for two sugarcane varieties (GT11 and GXB9) inoculated with CY4 and CN11 strains at 90 and 120 days was studied (Figure 8). The results showed that, using total RNA extracted from sugarcane leaf samples, *nifH* gene expression was positively detected by means of qRT-PCR. The highest expression of the *nifH* gene was recorded at 90 days, in GXB9 inoculated with strain CN11.

## Discussion

In this study, soil samples from sugarcane were used for the isolation of PGP nitrogen-fixing bacteria. Guangxi is the major sugarcane producing province in South China, and more than 60% of the total sugar production is from this area (Li and Yang, 2015). In China, the nitrogen application rate is very high, that is about 500–700 kg ha^−1^ annually for commercial sugarcane production and this is several times higher than in Brazil and other countries (Li et al., 2015). Higher application of nitrogen fertilizers not only raises the production cost, but also has an adverse effect on the environment and on soil health. Root-associated microbes have a positive effect on soil nutrient availability for plants (Glick et al., 1994). PGPR increases root surface area, increasing nutrient uptake and improving plant production (Mantelin and Touraine, 2004). Therefore, PGPR is an alternative method, the use of chemicals, for sugarcane nutrition because this crop requires large quantities of nitrogen fertilizer for growth and development. A number of nitrogen-fixing microbes have been reported in sugarcane (Xing et al., 2006, 2015; Mehnaz et al., 2009a; Lin et al., 2012; Solanki et al., 2017). *Pseudomonas* spp. are important PGPRs used as biofertilizer, and are able to enhance crop yield by direct and indirect mechanisms (Walsh et al., 2001), in addition to their characteristics of antibiotic production, phosphate solubilization, siderophore, IAA, HCN, and ammonia production (Ahemad and Khan, 2012a,b), nitrogen fixation, ACC deaminase activity, plant hormone production, and biological control. Of all the strains isolated in the present study, only 30 were selected on the basis of various different PGP traits, and nitrogenase activity.

Phosphorus is one of the most important plant nutrients and it greatly affects the growth of plants (Wang et al., 2009). In soil, P is highly insoluble and is therefore unavailable to plants. Phosphate-solubilizing microorganisms increase the performance of plants by providing them with soluble phosphorus. Phosphate solubilization has already been reported for different strains of *Pseudomonas frederiks-bergensis* (Zeng et al., 2016). In this study, we found that 26 (87%) isolates were positive for phosphate solubilization to convert insoluble tricalcium phosphate to the soluble form. Another important PGPR trait, which may indirectly influences plant growth, is the production of siderophores. Microorganisms play an important role in several essential biological processes and developed specific mechanisms for the assimilation of iron by production of low molecular weight iron-chelating compounds siderophores, which transport this element into their cells (Schwyn and Neilands, 1987; Arora et al., 2013). In the present investigation, 20 (66.67%) strains displayed siderophores production ability; siderophore production by *Pseudomonas* spp. is well known (Liu et al., 2011; Luo, 2014). HCN production by bacterial strains is important for diseases suppression and protection of plants from fungal diseases. HCN production by *P. fluorescens* strain CHA0 was related to biocontrol ability and root colonization; for example, suppression of tobacco black root rot caused by *Thielaviopsis basicola* (Voisard et al., 1989; Laville et al., 1992). The results of qualitative test of HCN production showed that 43.33% of the bacteria were capable of producing HCN. Volatile compounds, such as ammonia produced by a number of rhizobacteria, have been reported to play an important role in biocontrol (Brimecombe et al., 2001). Our results showed that 40.0% of the *Pseudomonas* strains were able to produce ammonia and might therefore play an important role in biocontrol activity. A previous isolate, *P. fluorescens* BAM-4, from semi-arid soil, is a potential biocontrol agent against *M. phaseolina* I mung bean and *P. aeruginosa* RM-3 showed lysis of several pathogenic fungi (Minaxi and Saxena, 2010a,b). To test strains for antifungal activity, we screened against two sugarcane pathogens (*U. scitaminea* and *C. paradoxa*); against these antagonistic activity was observed in 46.66 and 33.33% of strains, respectively (Table 2).

The role of ACC deaminase has been documented as one of the major mechanisms of PGP bacteria in promoting root and plant growth (Glick et al., 2007). Inoculation with ACC-deaminase containing bacteria promotes root growth of developing seedlings of various crops (Zahir et al., 2003). Inoculation with rhizobacteria having ACC deaminase activity resulted in the development of a better root system, which subsequently had a positive effect on shoot growth (Glick et al., 1998; Belimov et al., 2002). The screening of bacterial isolates obtained from sugarcane with DF medium and DF-ACC medium showed that 60% of the bacteria contained ACC deaminase, when grown on medium containing ACC as the sole nitrogen source. Most ACC-utilizing bacterial isolates are known to belong to genus *Burkholderia* and genus *Pseudomonas* (Blaha et al., 2006; Onofre-Lemus et al., 2009). All the selected bacterial strains showed ACC deaminase activity ranging from 77.0 to 15.13 μmoL mg^−1^ h^−1^, and higher activity was observed in strain CY4. In this study, we found that all bacterial strains were able to produce IAA in the range of 312.07–13.12 μg mL^−1^. The potential of the bacterial strains to produce IAA is indicative of their capability to be used as growth hormones or growth regulators. The results for nitrogen fixation as determined by ARA indicated that a large population of sugarcane-associated nitrogen-fixing bacterial strains is present in the soil and may be beneficial in improving the nitrogen level of sugarcane. The selected isolates were evaluated for their nitrogen-fixation efficiency, which was highly variable, ranging from 108.30 to 6.16 μmoL C_2_H_2_ h^−1^ mL^−1^. The incidence of nitrogen fixation in *Pseudomonas* spp. has been long debated, but recently several such *Pseudomonas* strains have been identified (Mirza et al., 2006). Several studies have also shown that nitrogenase producing bacteria can be isolated from sugarcane (Xing et al., 2006; Mehnaz et al., 2010; Lin et al., 2012; Solanki et al., 2017).

In this paper, we have also described a molecular approach for analyzing nitrogen-fixing genes in pure cultures isolated from sugarcane. Several primer have been used to amplify *nifH* genes, but after comparison of the sequences obtained from the *nifH* clones, we found the primer sets were is suitable for this study. All strains showed nitrogen-fixing activity by ARA in N-free medium, but the *nifH* gene was amplified only in 10 strains. *nifH* is one of the earliest characterized and best known functional genes (Rosado et al., 1998), and its amplification using degenerate primers is a useful tool for confirming nitrogen-fixation potential (Zehr and Capone, 1996). On the other hand, if amplification does not occur using the primers, this is not proof that strains are not proficient in nitrogen fixation, because the gene may show diverse nucleotide sequences between species and even within the same species (Zehr et al., 2003). An important objective of this study was to evaluate all the strains for antibiotic biosynthetic gene targets, because the production of various antimicrobial compounds is one of the biocontrol that helps to degrade pathogen cell walls and it is also an important factor for disease suppression (Sasirekha et al., 2013).

In the present study, we have demonstrated that repetitive extragenic sequences such as BOX, ERIC and REP are present in the genome of *Pseudomonas* bacteria. There little information published regarding the use of PCR to study the genetic diversity of *Pseudomonas* isolated from sugarcane. A method that was described for bacterial fingerprinting by examining strain specific banding patterns obtained from PCR amplification of repetitive DNA elements presented entire bacterial genomes (Versalovic et al., 1991). This technique is useful in the classification and differentiation of strains in many Gram-positive and Gram-negative bacteria, and also proved to be a powerful tool for initially screening within the strains. REP patterns are generally less complex than BOX and ERIC patterns, but all could give good discrimination at the strain level for the strains isolated from sugarcane (Figure 3). Our results also support a previous study in *P. aeruginosa* (Dawson et al., 2002), and the technique proved to be effective for evaluating the diversity of nitrogen-fixing bacterial strains.

Of all the strains, only two CY4 and CN11, were selected for further studies, such as Biolog profiling, GFP localization, and *nifH* gene expression, through qRT-PCR in sugarcane. Metabolic profiling of the selected isolates was performed using Biolog microplates, comprising analyses of the utilization of nutritional compounds (carbon and nitrogen), as well as tolerance to osmolytes and different pH conditions. The metabolic assets of an organism could contribute toward a particular adaptation and therefore might provide valuable information about bacteria supportive for root colonization (Mazur et al., 2013). The patterns of phenotypes obtained were suggestive that the selected strains were capable of utilizing a variety of metabolic substrates (Figure 5). Previously, it was suggested that more metabolically useful strains were more successful competitors in host plant nodulation (Wielbo et al., 2007). Remarkably, in our studies, the more metabolically diverse strain was CY4, rather than CN11. Through metabolic profiling, tolerance to osmolytes and different pH conditions was studied, using diversity indices such as Simpson 1-D, Shannon H, Evenness e^∧^H/S, Brillouin and Equitability J, and these methods revealed the substrate richness of the selected strains. Phenotypic profiling is important for understanding genotype differences, stress responses, media composition, and changes in environmental conditions for microorganisms (Chojniak et al., 2015).

We also investigated the effect of the selected *Pseudomonas* strains CY4 and CN11 on sugarcane by genetically tagging them with GFP-pPROBE-pTet^r^-OT and observing the level of colonization in plantlets. Both *Pseudomonas* strains colonized the whole plantlets as for roots, stems and leaves when inoculated separately. Uninoculated sugarcane plantlets served as the control and the plantlets did not show any appearance of fluorescence after 3 and 5 days of inoculation. This result showed that there was no fluorescence from whole plantlet tissues, but after comparison of strains CY4 and CN11, appeared that strain CY4 was a better colonizer for sugarcane plantlets. CLSEM and GFP have previously been used to demonstrate the colonization pattern of *Bacillus megaterium* in rice (Liu et al., 2006), *Klebsiella pneumoniae* in maize (Chelius and Triplett, 2000), *Rhizobium* sp. and *Burkholderia* sp. in rice (Singh et al., 2009), and *Microbacterium* sp. in sugarcane (Lin et al., 2012). This study shows that the GFP technique can be used effectively to evaluate competitive colonization capability for more than one plant-associated bacterium (Singh et al., 2009), or to select a potent strain isolated from different crops.

In, previous studies, it was considered that there were no nitrogen-fixing strains in the genus *Pseudomonas* (Setten et al., 2013). In fact, the inability of *Pseudomonas* spp. to fix nitrogen had been proposed as an important taxonomic character (Young, 1992; Anzai et al., 2000; Solanki et al., 2017). However, recent studies have confirmed that some strains belonging to the genus *Pseudomonas sensu stricto*, such as *P. stutzeri* A1501, *P. stutzeri* DSM4166, *P. azotifigens* 6HT33b^T^ and *Pseudomonas* sp. K1, have the capability to fix nitrogen (Mehnaz, 2011; Setten et al., 2013; Solanki et al., 2017). In our study, the selected *Pseudomonas* strains CY4 and CN11 also showed *nifH* gene expression in sugarcane, detected through qRT-PCR. Therefore, the presence of the *nifH* gene is indicative of the existence of diazotrophs, and the expression of the *nifH* gene is suggestive of the occurrence of BNF (Akter et al., 2014). The qRT-PCR technique is advantageous because of its high sensitivity and specificity, and detection of mRNA has been reported in ecological samples (Noda et al., 1999; Brown et al., 2003).

## Conclusion

To the best of our knowledge, this study is the first systematic report that has provided an awareness of the bacterial genus *Pseudomonas* associated with sugarcane rhizosphere. We have isolated bacteria that show useful activities in phosphate solubilization, siderophore production, ACC deaminase activity, and IAA-production, as well as N_2_-fixing activity and disease management. These features are measured as important PGP traits and have been found to be effective in improving the growth and nitrogen content of sugarcane plants. The strains CY4 (*P. koreensis*) and CN11 (*P. entomophila*) turned out to be very efficient in terms of enhancing the growth and development of plants, and disease control, as well as having nitrogenase activity. These organisms have greater potential to be used as biofertilizer due to their properties of nitrogen fixation, phytohormone production, and biocontrol capability. Assessment of these strains in field trials is now required, to determine their efficiency in plant growth promotion under field conditions. The inoculation of PGPR isolates may be an imminent development biofertilizer applications, for sustainable crop production, in reducing environmental pollution, and in biological agri-business.

## Author contributions

HL, RS, LY, and YL conceived and proposed the idea and drafted the manuscript. HL, RS, PS, QS, and YX carried out the experiments and conducted data analysis. All authors have read and approved the final manuscript.

### Conflict of interest statement

The authors declare that the research was conducted in the absence of any commercial or financial relationships that could be construed as a potential conflict of interest.
