# Supplementary material for: Genetic Diversity of Nitrogen-Fixing and Plant Growth Promoting Pseudomonas Species Isolated from Sugarcane Rhizosphere
Source: Front Microbiol. 2017 Jul 14;8:1268. doi: 10.3389/fmicb.2017.01268 (PMC5509769; doi:10.3389/fmicb.2017.01268)
Supplement: Supplementary file 1 [file Table1.DOCX]

**Table S1** The different media used in this study for isolation of bacteria from sugarcane field

| **S. No.** | **Media name and compositions (g L^-1^)** |
| --- | --- |
| 1 | **Ashbey’s Medium:** Mannitol; 15, CaCl_2_.2H_2_O; 0.2, MgSO_4_.7H_2_O; 0.2, MoO_3_ (10% solution); 0.1 mL, FeCl_3_ (10% solution); 0.05 mL, Agar; 15 |
| 2 | **Yeast Mannitol Agar Medium:** Mannitol; 15, K_2_HPO_4_; 0.5, Yeast Extract; 0.4, MgSO_4_.7H_2_O; 0.2, NaCl; 0.1, Agar; 15 |
| 3 | **LGI Medium:** Sucrose; 5, KH_2_PO_4_; 0.6; K_2_HPO_4_; 0.2; MgSO_4_.7H_2_O; 0.2, CaCl_2_.2H_2_O; 0.02, FeCl_3_; 1, Na_2_MoO_4_. 2H_2_O; 2 mg, Bromothymol Blue Solution; 5 mL, Agar; 1.75 |
| 4 | **Nutrient Agar:** Peptone; 5, NaCl; 5, Yeast Extract; 2, Beef Extract; 1, Agar; 15 |
| 5 | **DF** **salts minimal medium:** Glucose; 2, Gluconic acid; 2, Citric acid; 2, KH_2_PO_4_; 4, Na_2_HPO_4_; 6, MgSO_4_**.**7H_2_O; 0.2, Micro nutrient solution (in mg) (CaCl_2_; 200, FeSO_4_.7H_2_O; 200, H_3_BO_3_; 15, ZnSO_4_.7H_2_O; 20, Na_2_MoO_4_; 10, KI; 10, NaBr; 10, MnCl_2_; 10, COCl_2_; 5, CuCl_2_; 5, AlCl_3_; 2, NiSO_4_; 2. |
| 6 | **JNFb medium (Baldani et al. 1992):** Malic acid, 5.0; K_2_HPO_4_, 0.6; KH_2_PO_4_, 1.8; MgSO_4_.7H_2_O, 0.2; NaCl, 0.1; CaCl_2_.2H_2_O, 0.02.  Micronutrient solution (CuSO_4_.5H_2_O, 0.04; ZnSO_4_.7H_2_O, 0.12; H_3_BO_3_, 1.40; Na_2_MoO_4_.2H_2_O, 1.0; MnSO_4_. H_2_O, 1.175) (g L^-1^).  2 mL; bromothymol blue (5 g L^−1^ in 0.2 N KOH), 2 mL; Fe EDTA (16.4 g L^−1^), 4 mL; vitamin solution (biotin, 10 mg; pyridoxal-HCl, 20 mg, dissolved in hot-water bath. Complete to 100 mL by adding distilled water), 1 mL; KOH, 4.5g. Add distilled water to bring total solution to 1,000 mL. Adjust the pH to 6.8 with KOH. |

**Table S2** PCR conditions for amplification of genetic fingerprinting analysis in this study.

| **PCR conditions** | **Types of PCR** | | |
| --- | --- | --- | --- |
| **A. Reaction mixtures** | **BOX** | **ERIC** | **REP** |
| Taq-/Gitschier-buffer | 5 µL | 5 µL | 5 µL |
| BSA (20 mg mL^-1^) | 0.2 µL | 0.2 µL | 0.2 µL |
| DMSO (100%) | 2.5 µL | 2.5 µL | 2.5 µL |
| Taq polymerase (3 U) | 0.6 µL | 0.6 µL | 0.6 µL |
| dNTP mixture (100 mM mix) | 1.25 µL | 1.25 µL | 1.25 µL |
| Primer (Forward) | 1.3 µL (single primer) | 1.3 µL | 1.3 µL |
| Primer (Reverse) | **-** | 1.3 µL | 1.3 µL |
| Template DNA | 1.2 µL | 1.2 µL | 1.2 µL |
| Water (Molecular grade) | 12.95 µL | 11.65 µL | 11.65 µL |
| **B. Reaction conditions** | | | |
| Lead heat | 110°C | 110°C | 110°C |
| Initial Temperature | 94°C for 3 m | 95°C for 4 m | 95°C for 4 m |
| Start cycles | **-** | **-** | **-** |
| Number of cycles | 35 | 35 | 35 |
| Denaturations | 94°C for 30 s | 94°C for 1 m | 94°C for 1 m |
| Annealing | 50°C for 1 m | 52°C for 1 m | 40°C for 1 m |
| Elongation | 72°C for 8 m | 65°C for 8 m | 65°C for 8 m |
| End cycles | **-** | **-** | **-** |
| Final extensions | 72°C for 16 m | 65°C for 16 m | 65°C for 16 m |

**Table S3** Number of substrates utilized by individual strains.

| **Chemical Guild** | **Total number of Substrates** | **CY4** | **CY11** |
| --- | --- | --- | --- |
| Sugars | 27 | 20 | 13 |
| Chemical sensivity | 23 | 20 | 19 |
| Acidic pH | 2 | 2 | 2 |
| Sodium Chloride | 3 | 2 | 3 |
| Lactic acid | 1 | 1 | 1 |
| Hexose-PO_4_ | 2 | 1 | 1 |
| Amino acid | 9 | 7 | 8 |
| Hexose acid | 9 | 9 | 6 |
| Reducing Sugar | 2 | 2 | 2 |
| Carboxylic acids, esters and fatty acids | 18 | 13 | 14 |

**Table S4** Layout of Biolog Micro-Plate substrate present in each well.

| **Biolog well Serial No.** | **Carbon (GNIII)** | **Nitrogen (PM3B)** | **Osmolytes (PM9)** | **pH (PM10)** |
| --- | --- | --- | --- | --- |
| A1 | Negative Control | Negative Control | NaCl 1% | pH 3.5 |
| A2 | Dextrin | Ammonia | NaCl 2% | pH 4 |
| A3 | D-Maltose | Nitrite | NaCl 3% | pH 4.5 |
| A4 | D-Trehalose | Nitrate | NaCl 4% | pH 5 |
| A5 | D-Cellobiose | Urea | NaCl 5% | pH 5.5 |
| A6 | Gentiobiose | Biuret | NaCl 5.5% | pH 6 |
| A7 | Sucrose | L -Alanine | NaCl 6% | pH 7 |
| A8 | D-Turanose | L-Arginine | NaCl 6.5% | pH 8 |
| A9 | Stachyose | L-Asparagine | NaCl 7% | pH 8.5 |
| A10 | Positive Control | L - Aspartic Acid | NaCl 8% | pH 9 |
| A11 | pH 6 | L-Cysteine | NaCl 9% | pH 9.5 |
| A12 | pH 5 | L-Glutamic Acid | NaCl 10% | pH 10 |
| B1 | D-Raffinose | L-Glutamine | NaCl 6% | pH 4.5 |
| B2 | α-D-Lactose | Glycine | NaCl 6% + Betaine | pH 4.5 + L-Alanine |
| B3 | D-Melibiose | L-Histidine | NaCl 6% +N-N Dimethyl glycine | pH 4.5 + L-Arginine |
| B4 | β-Methyl-D-Glucoside | L-Isoleucine | NaCl 6% + Sarcosine | pH 4.5 + L- Asparagine |
| B5 | D-Salicin | L-Leucine | NaCl 6% + Dimethyl sulphonyl propionate | pH 4.5 + L- Aspartic Acid |
| B6 | N-Acetyl-D-Glucosamine | L-Lysine | NaCl 6% + MOPS | pH 4.5 + L- Glutamic Acid |
| B7 | N-Acetyl-β-DMannosamine | L-Methionine | NaCl 6% + Ectoine | pH 4.5 + L-Glutamine |
| B8 | N-Acetyl-D-Galactosamine | L- Phenylalanine | NaCl 6% + Choline | pH 4.5 + Glycine |
| B9 | N-AcetylNeuraminic Acid | L-Proline | NaCl 6% + Phosphoryl choline | pH 4.5 + L-Histidine |
| B10 | 1% NaCl | L-Serine | NaCl 6% + Creatine | pH 4.5 + L-Isoleucine |
| B11 | 4% NaCl | L-Threonine | NaCl 6% + Creatinine | pH 4.5 + L-Leucine |
| B12 | 8% NaCl | L-Tryptophan | NaCl 6% + L - Carnitine | pH 4.5 + L-Lysine |
| C1 | α-D-Glucose | L-Tyrosine | NaCl 6% + KCl | pH 4.5 + L-Methionine |
| C2 | D-Mannose | L -Valine | NaCl 6% + L-proline | pH 4.5 + L-Phenylalanine |
| C3 | D-Fructose | D-Alanine | NaCl 6% + N -Acethyl L-glutamine | pH 4.5 + L-Proline |
| C4 | D-Galactose | D-Asparagine | NaC1 6% + β-Glutamic acid | pH 4.5 + L-Serine |
| C5 | 3-Methyl Glucose | D-Aspartic Acid | NaC1 6% + γ –Amino -n -butyric acid | pH 4.5 + L-Threonine |
| C6 | D-Fucose | D-Glutamic Acid | NaC1 6% + Glutathione | pH 4.5 + L-Tryptophan |
| C7 | L-Fucose | D-Lysine | NaCl 6% + Glycerol | pH 4.5 + L-Tyrosine |
| C8 | L-Rhamnose | D-Serine | NaC1 6% + Trehalose | pH 4.5 + L-Valine |
| C9 | Inosine | D-Valine | NaC1 6% + Trimethylamine -N-oxide | pH 4.5 + Hydroxy- L-Proline |
| C10 | 1% Sodium Lactate | L-Citrulline | NaC1 6% + Trimethylamine | pH 4.5 + L-Ornithine |
| C11 | Fusidic Acid | L-Homoserine | NaCl 6% + Octopine | pH 4.5 + L-Homoarginine |
| C12 | D-Serine | L-Ornithine | NaC1 6% + Trigonelline | pH 4.5 + L-Homoserine |
| D1 | D-Sorbitol | N - Acetyl-D,L-Glutamic Acid | Potassium chloride 3% | pH 4.5 + Anthranilic acid |
| D2 | D-Mannitol | N-Phthaloyl-L Glutamic Acid | Potassium chloride 4% | pH 4.5 + L-Norleucine |
| D3 | D-Arabitol | L-Pyroglutamic Acid | Potassium chloride 5% | pH 4.5 + L-Norvaline |
| D4 | myo-Inositol | Hydroxylamine | Potassium chloride 6% | pH 4.5 + L-α- Amino - N- butyric acid |
| D5 | Glycerol | Methylamine | Sodium sulfate 2% | pH 4.5 + L- p - Aminobenzoate |
| D6 | D-Glucose-6-PO4 | N-Amylamine | Sodium sulfate 3% | pH 4.5 + L- Cysteic acid |
| D7 | D-Fructose-6-PO4 | N-Butylamine | Sodium sulfate 4% | pH 4.5 + D-Lysine |
| D8 | D-Aspartic Acid | Ethylamine | Sodium sulfate 5% | pH 4.5 + 5-Hydroxy Lysine |
| D9 | D-Serine | Ethanolamine | Ethylene glycol 5% | pH 4.5 + 5-Hydroxy Tryptophan |
| D10 | Troleandomycin | Ethylenediamine | Ethylene glycol 10% | pH 4.5 + D,L-Diamino pimelic acid |
| D11 | Rifamycin SV | Putrescine | Ethylene glycol 15% | pH 4.5 + Trimethyl amine-N-oxide |
| D12 | Minocycline | Agmatine | Ethylene glycol 20% | pH 4.5 + Urea |
| E1 | Gelatin | Histamine | Sodium formate 1% | pH 9.5 |
| E2 | Glycyl-L-Proline | β-Phenylethyl-amine | Sodium formate 2% | pH 9.5 + L-Alanine |
| E3 | L-Alanine | Tyramine | Sodium formate 3% | pH 9.5 + L-Arginine |
| E4 | L-Arginine | Acetamide | Sodium formate 4% | pH 9.5 + L-Asparagine |
| E5 | L-Aspartic Acid | Formamide | Sodium formate 5% | pH 9.5 + L-Aspartic Acid |
| E6 | L-Glutamic Acid | Glucuronamide | Sodium formate 6% | pH 9.5 + L-Glutamic Acid |
| E7 | L-Histidine | D,L-Lactamide | Urea 2% | pH 9.5 + L-Glutamine |
| E8 | L-Pyroglutamic Acid | D-Glucosamine | Urea 3% | pH 9.5 + Glycine |
| E9 | L-Serine | D-Galactosamine | Urea 4% | pH 9.5 + L-Histidine |
| E10 | Lincomycin | D-Mannosamine | Urea 5% | pH 9.5 + L-Isoleucine |
| E11 | Guanidine HCl | N-Acetyl-D-Glucosamine | Urea 6% | pH 9.5 + L-Leucine |
| E12 | Niaproof 4 | N-Acetyl-D-Galactosamine | Urea 7% | pH 9.5 + L-Lysine |
| F1 | Pectin | N-Acetyl-D-Mannosamine | Sodium Lactate 1% | pH 9.5 + L-Methionine |
| F2 | D-Galacturonic Acid | Adenine | Sodium Lactate 2% | pH 9.5 + L-Phenylalanine |
| F3 | L-Galactonic Acid Lactone | Adenosine | Sodium Lactate 3% | pH 9.5 + L-Proline |
| F4 | D-Gluconic Acid | Cytidine | Sodium Lactate 4% | pH 9.5 + L-Serine |
| F5 | D-Glucuronic Acid | Cytosine | Sodium Lactate 5% | pH 9.5 + L-Threonine |
| F6 | Glucuronamide | Guanine | Sodium Lactate 6% | pH 9.5 + L-Tryptophan |
| F7 | Mucic Acid | Guanosine | Sodium Lactate 7% | pH 9.5 + L-Tyrosine |
| F8 | Quinic Acid | Thymine | Sodium Lactate 8% | pH 9.5 + L-Valine |
| F9 | D-Saccharic Acid | Thymidine | Sodium Lactate 9% | pH 9.5 + Hydroxy- L-Proline |
| F10 | Vancomycin | Uracil | Sodium Lactate 10% | pH 9.5 + L-Ornithine |
| F11 | Tetrazolium Violet | Uridine | Sodium Lactate 11% | pH 9.5 + L-Homoarginine |
| F12 | Tetrazolium Blue | Inosine | Sodium Lactate 12% | pH 9.5 + L-Homoserine |
| G1 | p-Hydroxy- Phenylacetic Acid | Xanthine | Sodium Phosphate pH 7 20mM | pH 9.5 + Anthranilic acid |
| G2 | Methyl Pyruvate | Xanthosine | Sodium Phosphate pH 7 50mM | pH 9.5 + L-Norleucine |
| G3 | D-Lactic Acid Methyl Ester | Uric Acid | Sodium Phosphate pH 7 100mM | pH 9.5 + L-Norvaline |
| G4 | L-Lactic Acid | Alloxan | Sodium Phosphate pH 7 200mM | pH 9.5 + Agmatine |
| G5 | Citric Acid | Allantoin | Sodium Benzoate pH 5.2 20mM | pH 9.5 + Cadaverine |
| G6 | α-Keto-Glutaric Acid | Parabanic Acid | Sodium Benzoate pH 5.2 50mM | pH 9.5 + Putrescine |
| G7 | D-Malic Acid | D,L-α-Amino-N-Butyric Acid | Sodium Benzoate pH 5.2 100mM | pH 9.5 + Histamine |
| G8 | L-Malic Acid | γ-Amino-N-Butyric Acid | Sodium Benzoate pH 5.2 200mM | pH 9.5 + Phenylethylamine |
| G9 | Bromo-Succinic Acid | ε-Amino-N-Caproic Acid | Ammonium sulfate pH8 10mM | pH 9.5 + Tyramine |
| G10 | Nalidixic Acid | D,L-α-Amino-Caprylic Acid | Ammonium sulfate pH8 20mM | pH 9.5 + Creatine |
| G11 | Lithium Chloride | δ-Amino-N-Valeric Acid | Ammonium sulfate pH8 50mM | pH 9.5 + Trimethyl amine- N-oxide |
| G12 | Potassium Tellurite | α-Amino-N-Valeric Acid | Ammonium sulfate pH8 100mM | pH 9.5 + Urea |
| H1 | Tween 40 | Ala-Asp | Sodium Nitrate 10mM | X-Caprylate |
| H2 | γ-Amino-ButryricAcid | Ala-Gln | Sodium Nitrate 20mM | X–α-D-Glucoside |
| H3 | α-Hydroxy- Butyric Acid | Ala-Glu | Sodium Nitrate 40mM | X-β-D-Glucoside |
| H4 | β-Hydroxy-D,LButyricAcid | Ala-Gly | Sodium Nitrate 60mM | X-α-D-Galactoside |
| H5 | α-Keto-Butyric Acid | Ala-His | Sodium Nitrate 80mM | X-β-D-Galactoside |
| H6 | Acetoacetic Acid | Ala-Leu | Sodium Nitrate 100mM | X-α-D-Glucuronide |
| H7 | Propionic Acid | Ala-Thr | Sodium Nitrite 10mM | X-β-D-Glucuronide |
| H8 | Acetic Acid | Gly-Asn | Sodium Nitrite 20mM | X-β-D-Glucosaminide |
| H9 | Formic Acid | Gly-Gln | Sodium Nitrite 40mM | X-β-D-Galactosaminide |
| H10 | Aztreonam | Gly-Glu | Sodium Nitrite 60mM | X-α-D-Mannoside |
| H11 | Sodium Butyrate | Gly-Met | Sodium Nitrite 80mM | X-PO4 |
| H12 | Sodium Bromate | Met-Ala | Sodium Nitrite 100mM | X-SO4 |
